# Subtle differences in family size mediate object play in young ravens (*Corvus corax*)

**DOI:** 10.1038/s42003-026-10290-0

**Published:** 2026-07-11

**Authors:** Palmyre H. Boucherie, Valentine Comin, Thomas Bugnyar

**Affiliations:** 1 Department of Behavioural and Cognitive Biology, University of Vienna, Vienna, Austria; 2 Haidlhof Research Station, University of Vienna and University of Veterinary Medicine Vienna, Bad Vöslau, Austria; 3 Konrad Lorenz Research Center, University of Vienna, Grünau, Austria

**Keywords:** Animal behaviour, Behavioural ecology

## Abstract

The social environment at upbringing shapes the developmental trajectories of offspring behaviour and competences in various species. Surprisingly, little is known about how slight variations in early social environments, such as differences in sibling numbers, affect these trajectories. Common ravens undergo an impressive socio-cognitive development post-fledging, engaging in various forms of play. We investigate the influence of (manipulated) family size on object-directed behaviours of juvenile ravens, testing the prediction that larger family size would foster more object play at standardised playgrounds. Contrary to our prediction, family size does not affect the individuals’ access to objects and amount of play; however, offspring from small families are more often together at the playground and show more and selective interest in others’ object manipulations than offspring from large families. Our findings indicate that even slight differences in family size – within natural range - can mediate juveniles’ opportunities to interact, play, and develop their social repertoire.

## Abstract (web summary)

The social environment at upbringing influences offspring’s social experience and competence development across species. In ravens, even minor differences in family size affect juveniles’ opportunities to interact with others around objects.

## Introduction

Life in structured social groups is considered cognitively demanding, as individuals need to adjust their decisions to conspecific behaviour, recognise other group members and track social relationships^1,2^. Developing the ability to optimise one’s behaviour based on available social information, known as social competence^3^, may be essential to reduce the cognitive demands of group living. Previous research has shown that the early social environment can have a long-lasting effect on social competence^4–6^. For instance, increased group size (e.g. communal rearing^5,7,8^, enlarged groups^9^) and dynamic group composition^10^ may enhance social competence later in life, likely because a variable social environment creates ample learning opportunities about conspecifics^11^. However, increased group size may also increase competition for resources and thus stress during development, which can alter the functioning of the stress response later in life^12,13^ and the expression of social behaviours^14–16^.

The described effects of early social environment rely on experimental manipulations during upbringing, but mostly, it remains unclear how those translate into ecologically valid scenarios in natural settings. For instance, studies often focus on the effects of social deprivation or artificially altered group composition, either inflated or atypical (e.g. in rodents^4,17,18^, fish^19,20^, primates^21,22^ and birds^23,24^). Such approaches often diverge from the range of natural early-life conditions encountered by the species (but see ref. ^25^ for a study on wild zebra finches and the consequences of brood size manipulations on patterns of spatial association). Furthermore, most studies explored the consequences of specific treatments early in life on competencies later in life. In contrast, only very few studies report how the social experiences vary during the treatment^11^. Hence, instead of solely examining the link between competencies and extreme early-life conditions, it is crucial to explore the natural variations in rearing environments and assess how these differences influence the social experience of juveniles.

To explore this, an interesting candidate among birds is the common raven (*Corvus corax*), known for its complex social behaviour and advanced cognitive skills^26–29^. After leaving the family, juveniles join socially dynamic non-breeder groups^30^, where they form affiliative relationships that may eventually develop into long-term monogamous pair bonds^31–33^. Because this non-breeder phase is already socially demanding, the earlier social experiences within the family unit likely contribute to shaping individual social skills. A naturally varying aspect of the early social environment in birds is brood size. In common ravens, both parents provide care for up to four or five months to broods ranging from one to six chicks^31^. However, brood sizes are typically centred around two to four chicks in captivity and in the wild: for instance, Ratcliffe^31^ reported a mean clutch size of 4.8 eggs in the UK, with fledged brood sizes typically ranging from 2.2 to 4.2, depending on location. Only 10% of broods reached five fledglings, and 1% reached six. Similarly, reported fledgling success in Switzerland ranged between 1.2 and 2.4 young per nest^34^. These data indicate that brood sizes in natural settings remain within a narrow range. To accurately reflect the natural variation observed in wild populations, it is thus essential to examine subtle differences in brood size. If early-life conditions shape social competences in ravens, and if brood size serves as a good proxy for those conditions, then even small variations in brood size may have measurable effects on developmental experiences and later social behaviour.

In this study, we used a play context to explore if and how variations in brood size affect the social experience of juvenile ravens within their family unit. We focused on object play, defined as spontaneous, repeated, and non-functional object manipulations^35^. By offering sets of wooden objects outside feeding (i.e. in a relaxed atmosphere), we aimed to elicit object-directed behaviours at a standardised playground and social interactions around objects (including co-occurrence at the playground and showing interest in others’ object manipulation). Captive ravens engage in playful object manipulation as early as the nestling stage^36^, with play increasing after fledging and declining in adults^37^, a pattern recently observed in wild individuals as well^38^. Juvenile ravens are highly neophilic, preferring novel over familiar objects^39^. They also show selective attention to their siblings^40^, and their likelihood to interact with an object increases after watching a sibling playfully manipulate it^41^. Watching others engage with objects may also trigger general play activities—including social and locomotory play—due to emotional contagion^36,42^. Finally, young ravens are renowned for playfully caching inedible objects in the presence of conspecifics, a behaviour thought to help develop cache protection strategies^43^ and test unfamiliar individuals’ pilfering skills^44^ without the cost of losing valuable food items. These learning aspects of object play might be especially relevant for developing social competence, notably for individuals with multiple siblings. The variety of ways ravens interact with objects and their functions later in life—e.g. efficient food caching and social bonding—suggests that early interactions and learning about objects are crucial for developing social competencies.

Here, we tested 11 captive raven families in an object play experiment. Per family, we experimentally manipulated the offspring number at the egg level, without affecting the relatedness between offspring, creating large families with three offspring and small families with one or two offspring. This design allowed us to examine the effects of subtle, ecologically relevant variations in brood size. After fledging, families were tested in weekly blocks with standardised sets of objects, each including familiar and novel items. Test blocks were replicated three times over a nine-week period. Given the contagious nature of object play^36,42^, we expected offspring from large families to spend more time engaging in object play than those from small families. Given the rapid socio-cognitive development after fledging^41,43,45,46^, we expected both object play and patterns of interactions between family members to change across the weekly blocks, whereby the family size could mediate the strength of the effects. Given the neophilic nature of juvenile ravens, we expected the objects’ novelty to affect their manipulation (positively), regardless of family size.

## Materials and methods

### Animals and housing

We tested 25 juvenile ravens (*Corvus corax*) in 11 families between 2019 and 2021. The 11 families originated from six captive breeding pairs that reproduced over multiple years. Following the number of offspring per year, families were characterised as large (F+: three offspring, *n* = 5) or small (F−: one to two offspring, *n* = 6; Supplementary Table 1). The number of offspring was manipulated by removing eggs from the nest, resulting in four eggs for F+ and two for F−. Which eggs were removed was determined randomly and did not follow the laying order. Note also that our brood manipulation did not alter the relatedness among the chicks, as we typically did not exchange eggs between breeding pairs (with the exception of one pair, Supplementary Table 1), and we never mixed eggs of different clutches. In some nests, not all chicks hatched and/or survived the first days, resulting in the final numbers of one to three offspring per pair (Supplementary Table 1). Within pairs, the family size switched across the years. In all families, the offspring stayed with their parents until mid-July (four months post-hatch). All offspring were then moved to non-breeder aviaries, remaining for about two months before being allowed into free flight.

The families were housed in separate aviaries located at three sites: (a) Cumberland Wildpark, Grünau im Almtal; (b) Haidlhof Research Station, Bad Vöslau and (c) Tiergarten Schönbrunn, Zoo Vienna (Supplementary Table 1). The aviaries at the first two sites are similar in size (about 80 m²); the aviary at Zoo Vienna is about double the size (~160 m²). All aviaries were enriched with wood and metal perches, trees and rocks, pool, gravel, grass, ropes, small toys and other objects (e.g. shoes, empty bottles). Birds were provided water ad libitum and food twice a day, in the morning and in the afternoon. Meals comprised meat and fresh products (e.g. fruits, vegetables, raisins and grains). For individual identification, all ravens were marked with coloured leg rings.

We have complied with all relevant ethical regulations for animal use. The housing and testing conditions comply with the ASAB/ABS Guidelines for the Use of Animals in Research, the Austrian government guidelines, and the institutional guidelines of the University of Vienna. Permission for keeping ravens and for releasing the offspring of captive ravens into free flight was granted by the Austrian Ministry for Education, Science and Research (updated GZ 2021-0.391.938) and the BH Gmunden (Ausnahmebewilligung §29 Oö. NSchG 2001; valid from 2018–2028), respectively. The study was approved by the Animal Ethics and Experimentation Board of the Faculty of Life Science, University of Vienna (permit number 20-003). As this study was strictly non-invasive and based purely on behavioural tests, it is not classified as an animal experiment under the Austrian Animal Experiments Act (§2. Federal Law Gazette No. 501/1989). All ravens participated in the experiment voluntarily, were never deprived of food or water, and were tested in their own aviaries.

### Experimental settings

The experiment was conducted between May and June, starting as soon as the offspring had fledged (typically during the first week of May). Testing spanned three weekly blocks over nine weeks, allowing us to capture developmental (temporal) effects in our analyses. Each weekly block included test sessions conducted across four days, with one session in the morning and one in the afternoon, resulting in a maximum of eight test sessions per week and 24 test sessions per family per year. Due to the COVID-19 pandemic, two families in 2020 were tested for only two weeks, completing 16 test sessions as they missed the first test block. Additionally, one family missed two sessions during the first test block (see Supplementary Table 1).

Each test session lasted 15 min and started when the two boards containing the objects were provided as a ‘playground’ to the family group inside the aviary. Experiments were conducted by groups of students from the University of Vienna (see the ‘acknowledgement’ section for more details) and with the help of the animal keepers on site. All experiments were recorded using a hand-held video camera (focusing on interactions and recording verbal comments of the experimenter) and a global camera (providing an overview of the aviary) (see Supplementary Note 1 for more details). The two different angles were combined in one single multiple-view video for scoring, using the video editing software Blackmagic Design DaVinci Resolve 16 (Blackmagic Design, 2019).

**Fig. 1:**
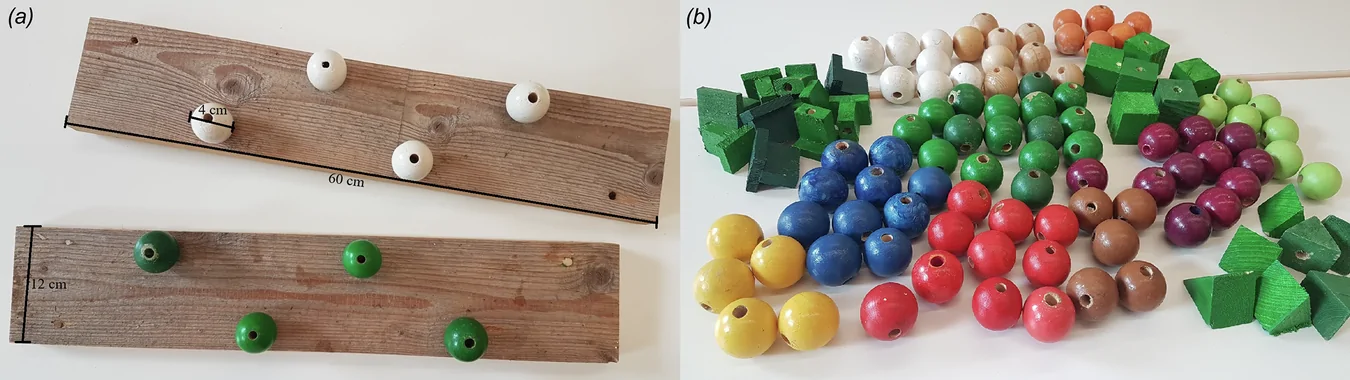
Playground apparatus. Playground apparatus with (**a**) the familiar board at the bottom and the novel board with the ‘white balls’ condition at the top, and **b** the different objects used in 2021. The material and size of the objects were chosen based on pilot studies, in which wooden items of approximately that size elicited moderate manipulation rates but were regularly attended to by adult and juvenile ravens.

The set-up consisted of two wooden boards with painted wooden objects on top (Fig. 1). The number of objects per board remained constant across test sessions within a family and was determined by the number of individuals in the family, resulting in three to five objects per board (one for each parent and one for each offspring). Note that the position of the objects on the boards was standardised so that the distance between items remained the same, irrespective of their number (Fig. 1a). We refrained from adjusting the length of the board to the number of objects and the size of the area covered by our set-up (e.g. by having multiple small boards), because ravens are very sensitive to changes in the environment^47^ and we wanted to keep the set-up as similar and simple as possible when experimentally working with families.

One board always contained ‘familiar’ objects (dark green balls), which remained the same for all test sessions and families, while the second contained ‘novel’ objects, which changed every day. The novel objects differed from the familiar objects by one attribute (colour or shape, but not material or size; Fig. 1b). Familiar objects were introduced to the families 2 or 3 days before the first test session. Half of the objects, familiar and novel ones alike, were fixed to the board, whereas the other half rested on a wooden dowel and could easily be removed by the ravens (family of three: two out of three objects per board were removable; family of four: two out of four objects per board were removable; family of five: three out of five objects per board were removable). Presenting fixed and movable objects on a board should allow for different types of object play. It should also reduce the likelihood of birds crowding at one board, as individuals could simply take an object and leave.

### Behavioural response scoring

We used behavioural sampling to score the 15-min test sessions, using Loopy coding software for quantitative video analysis from Loopbio (http://loopb.io, loopbio gmbh, Vienna, Austria; screenshot of the coding platform on Supplementary Fig. 1). We coded all individual behaviours and social interactions between family members within a 2-m radius around the object playground (i.e. zone 2). Interactions outside this zone were ignored. Additionally, we defined a smaller inner zone (zone 1), spanning 50 cm around the object playground, to evaluate the time individuals spent near the object set-up and their proximity to one another around the playground. This area was designated by the coder within the video using scaling elements in the aviary to prevent ground marking and minimise potential disturbance to the birds. Note that the entire zone 1 is slightly larger than 1 m: 50 cm on each side, plus the space occupied by the two boards (which could be moved by the ravens). For all behaviours, we coded the nature of the behaviour, the duration when relevant, and the identity of both partners for social interactions and the directionality of the interaction.

The present analyses focused on the following behaviours: (i) presence in zone 1; (ii) object manipulations (i.e. manipulating, caching/retrieving from a cache, or transporting an object); (iii) proximity with another individual within zone 1 (individuals being 0–1 m apart while in zone 1); and (iv) showing interest in another individual manipulating or transporting an object (i.e. watch or follow another individual manipulating an object or following an individual who is transporting an object). See the Supplementary Table 2, for a detailed ethogram of the response variables used in the modelling approach.

Additionally, we descriptively analysed the order of access to the playground at each session. We focus, in particular, on the first individual to access the playground, defined as the first individual to enter zone 1 around the apparatus.

We also conducted a descriptive analysis of how family members accessed the playground and its objects across family sizes. Specifically, we examined: (i) the frequency of agonistic interactions (i.e. avoid, charge, chase, fight, peck, supplant, threat); (ii) the frequency of object removal from a board; (iii) the frequency of object transport away from the playground (i.e. outside zone 2); (iv) the count of unique individuals accessing the playground; (v) the count of unique individuals manipulating an object or a board. Data were summarised: per family unit and test session, and normalised by the number of dyads (i); or per age class (adult vs. chick) within each family unit and test session, and normalised by the number of individuals per age class (ii–v). Finally, we examined the total time and mean bout duration spent in zone 1, manipulating objects, and standing on a board (i.e. one individual with one or both feet on a board), per individual and per test session.

### Statistics and reproducibility

We analysed the individual behaviour and social experience of offspring across four object-related variables: (i) individual access to the object playground, computed as the proportion of the time spent within zone 1 during the 15 min experiment (*N* = 68 observations, data aggregated per individual [24, with known sex] and test block [3]; four values missing due to two families with missing data in test block one); (ii) individual object manipulation, computed as the proportion of the time manipulating objects during the 15 min experiment (*N* = 136 observations, data aggregated per individual, test block and object novelty [2], doubling the number of observations compared to (i)); (iii) dyadic frequency of proximity in zone 1, defined as the number of instances in which an offspring either initiated or received proximity with any other family member near the playground (*N* = 196 observations, data aggregated per dyad [69] and test block; 11 values missing); (iv) the dyadic frequency of showing interest in another individual manipulating an object, defined as the number of instances where an offspring directed attention to or followed any family member engaging in object manipulation (*N* = 392 observations, data aggregated per dyad, test block and object novelty; doubling the number of observations compared to (iii)).

For ‘showing interest’, analyses were restricted to interactions initiated by offspring, which accounted for 96% of all cases (58% directed towards a parent and 42% towards a sibling), whereas only 4% were initiated by parents. Our focus thus aligns with the typical distribution of this behaviour in families at this stage and allows us to examine the factors mediating chicks’ interest. For proximities, offspring initiated 78.6% of all cases (36% towards a parent and 64% towards a sibling), while parents initiated 17.7%, primarily toward offspring (67% vs. 33% toward their partner). A small fraction of interactions (3.7%) had no identifiable initiator. Because interactions involving at least one offspring, either as initiator or receiver, accounted for 90% of all proximity events, analyses focused on this subset (all proportions were extracted from this dataset).

We ran four generalised linear mixed models (GLMMs): the first two relating to offspring behaviour, using beta distributions with log-link functions for the proportion of time spent around the object playground and the proportion of object manipulation (function ‘glmmTMB’, glmmTMB R package version 1.1.5^48^); and the two other models relating to offspring social experience, using negative binomial distributions and log-link functions for the frequencies of proximity and ‘showing interest’ to account for their over-dispersed distributions (function ‘glmmTMB’, family: nbinom2 or nbinom1). We included the number of observation sessions in offset for the frequency models since one family was tested six times instead of eight in the first test block. Fixed factors included family size (small: one to two offspring vs. large: three offspring), test block (categorical: one, two, three), individual sex or dyad age (siblings, offspring-parent), and for the object manipulation and the ‘showing interest’ models, the novelty of the object (novel vs. familiar). We also considered the interactions between family size and test block, family size and sex or dyad age, and family size and object novelty (when relevant).

Not all families contributed to every level of the factors sex or dyad age due to family composition, such as single-chick or single-sex-offspring families. Although this does not bias the overall effect of sex or dyad age across the dataset, it should be recognised as a structural limitation of the design, rather than a sampling or measurement error. As a result, effects are estimated across families rather than within each family. However, to verify that the results were robust to the inclusion or exclusion of this particular family composition, we conducted a sensitivity analysis by rerunning all models after excluding single-chick families.

We also added the location of the aviary (Cumberland Wildpark, Vienna area) as a control fixed factor. Because only a single family was housed at Zoo Vienna, limiting within-site replication, we combined this location with the nearby Haidlhof research station under a common ‘Vienna valley’ category to prevent model singularity. Both sites experience comparable climatic conditions, distinct from the alpine environment of the Cumberland Wildpark. Importantly, all facilities applied consistent standards for animal care, housing, and experimental procedures. The birds were also similarly habituated to human presence across sites, whether from researchers, students, or public visitors, helping to minimise behavioural differences related to site exposure. To account for non-independence of observations due to repeated measures and to control for potential individual or dyad-level variance, we included individual or dyad identity as a random intercept. These were nested within family identity to control for shared genetic background or parental influence. For the model assessing access to the playground, this nested structure was simplified (individual identity only) to improve model fit and avoid singularity. We also attempted to include all applicable random slopes for all models, but they consistently caused model singularity and were subsequently removed.

We tested each interaction’s overall significance with full–reduced model comparisons between the full model and the reduced models lacking the interaction of interest. We also ran full null model comparisons between the full model and the null model, which lacked all fixed factors (but included the random factors). Model comparisons were performed with the anova R test function, from which we reported AIC, degree of freedom, χ2 and *p*-values. All our analyses were conducted in R software v. 4.4.2. with a significance threshold set at *α* = 0.05.

### Reporting summary

Further information on research design is available in the Nature Portfolio Reporting Summary linked to this article.

## Results

Throughout the study, in six small (one to two offspring) and five large (three offspring) families, we monitored 25 offspring (24 in models investigating offspring’s behaviour). Regarding offspring behaviour, we recorded a total of 1045 events at the playground (compared to 474 for the 22 parents), equivalent to 810 min spent around the playground (vs. 388 min for parents; Supplementary Fig. 7). We also recorded 3819 object manipulations by offspring (vs. 1997 for parents), equivalent to 411 min spent around the playground (vs. 185 min for parents; Supplementary Fig. 8). Regarding offspring social experience, we recorded 704 proximity events emitted or received by offspring (vs. 48 between parents) and 303 instances of ‘showing interest’ emitted by the offspring towards siblings or parents (vs. 134 emitted by a parent).

### Behaviour of offspring

#### Time spent near the playground

**Fig. 2:**
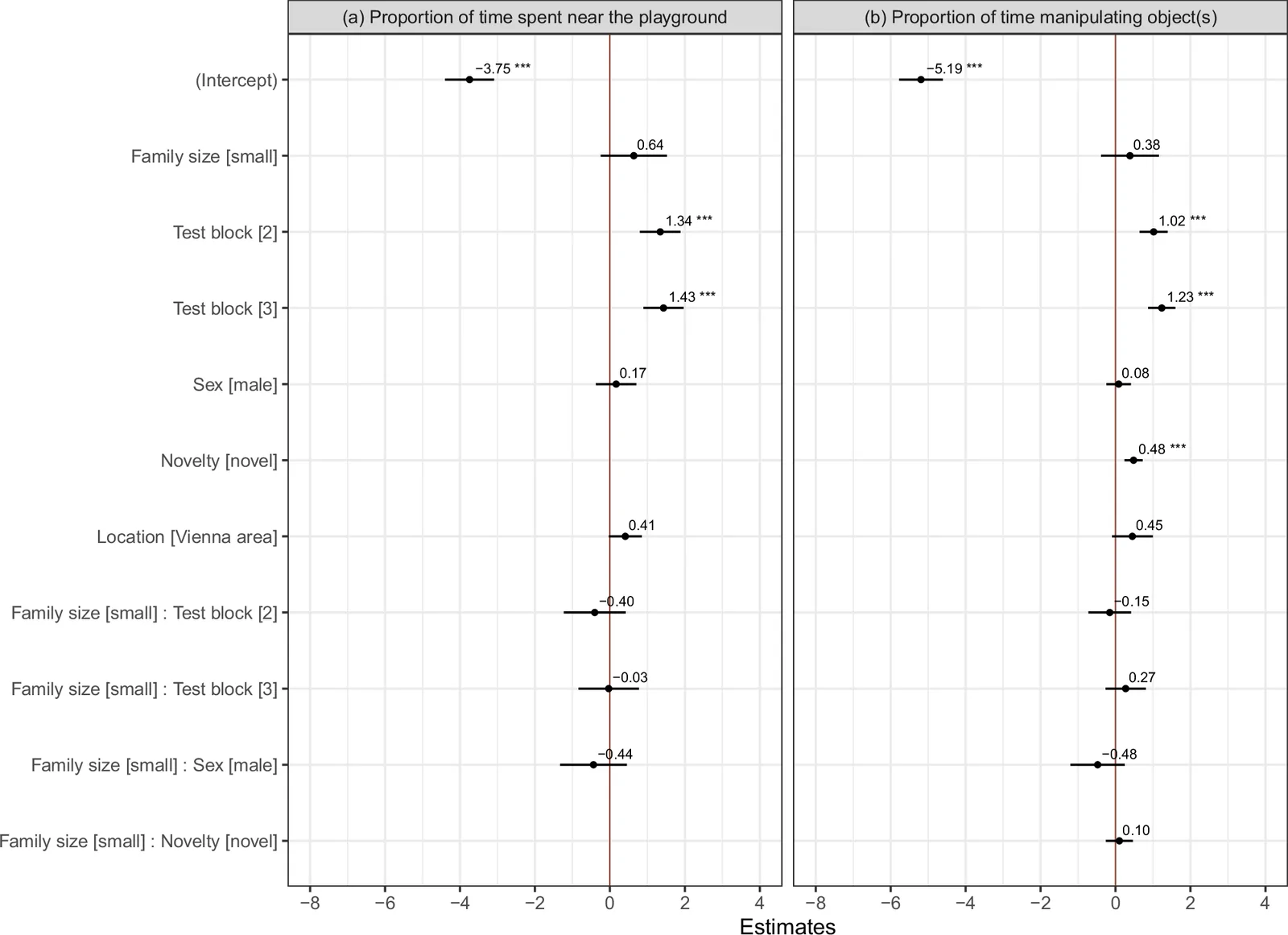
Model estimates of offspring engagement with the playground area and objects. Estimates forest plots for the models investigating (**a**) the individual proportion of time spent near the object playground (i.e. within a 50-cm round zone around the playground) and **b** the individual proportion of time manipulating object(s), computed per offspring and test block. Reference levels for the categorical variables are: family size (large), test block (1), sex (female), novelty (familiar) and Location (Cumberland Wildpark). Points represent model estimates, and error bars represent 95% confidence intervals. Sample size: **a** *N* = 68 observations, data aggregated per individual (*n* = 24, with known sex) and test block (*n* = 3), four values missing due to two families with missing data in test block one; **b** *N* = 136 observations, data aggregated per individual, test block and object novelty (*n* = 2), doubling the number of observations compared to (**a**). See Supplementary Tables 3 and 5 for the full model outputs. ***<0.001, **<0.01, *≤0.05.

**Fig. 3:**
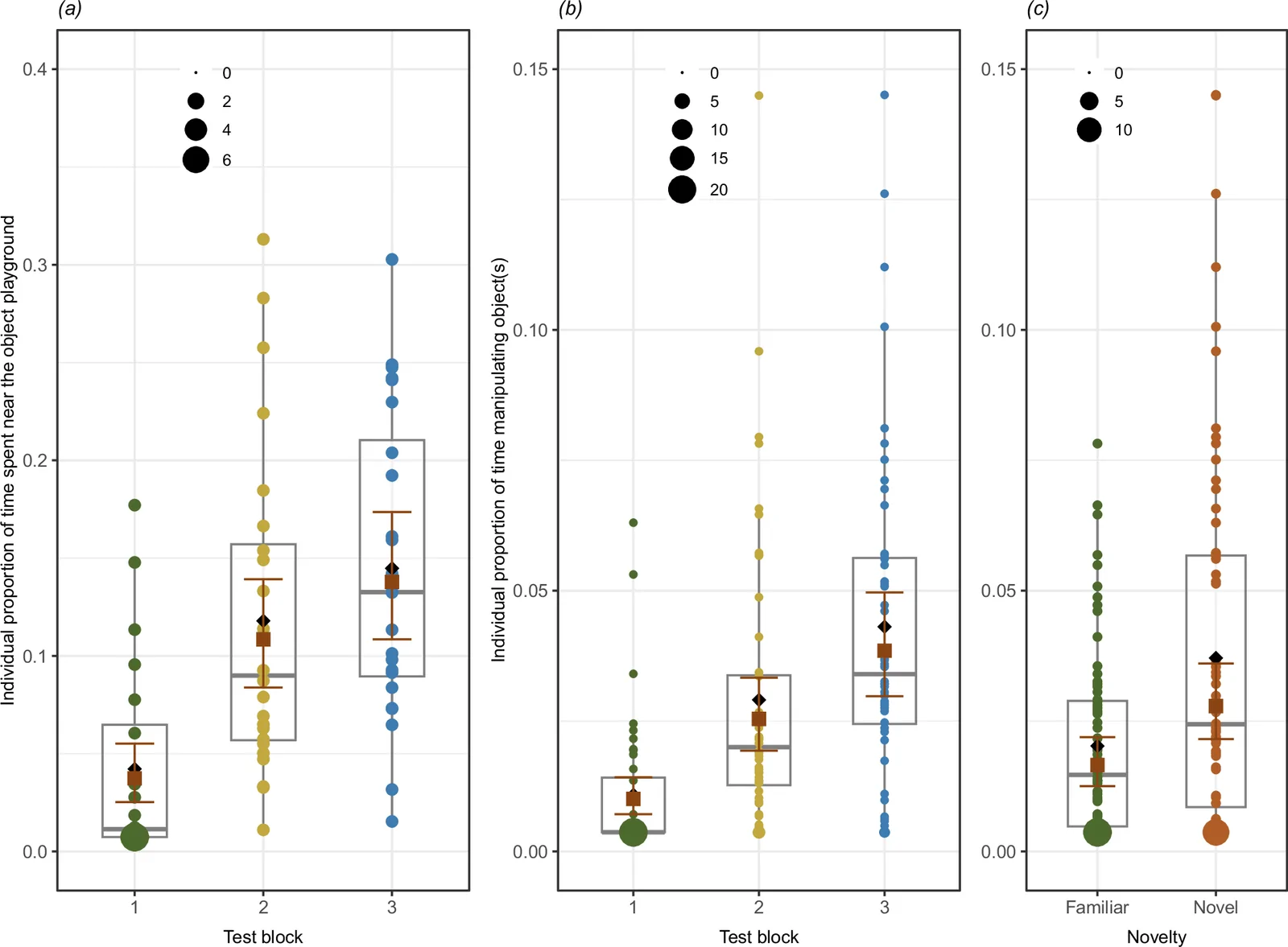
Raw data and model predictions of offspring engagement with the playground area and objects across the test block and object novelty. Raw data and model predictions for: **a** the individual proportion of time spent near the object playground; **b**, **c** the individual proportion of time manipulating object(s); according to the test block (**a**–**b**), and the object novelty (**c**). Proportions were computed per offspring and test block. Plots display raw data (dot size proportional to the number of observations) alongside: boxplots (showing the median and interquartile range), raw data means (black diamonds), and model predictions (brown square, estimated marginal means generated with the predict_response function, ggeffects R package) with 95% confidence intervals (error bars). The plots were generated using ggplot2.

The test block significantly influenced the proportion of time offspring spent near the playground (Fig. 2a and Supplementary Table 3). Regardless of family size, offspring spent more time near the playground as the experiment progressed and they aged, with a sharper increase between test blocks one and two compared to blocks two and three (Fig. 3a).

This effect was robust to model specification (see sensitivity check for singleton families; Supplementary Fig. 2 and Supplementary Table 4). When families with only one chick were excluded, model results remained largely consistent, except that a new significant interaction between family size and test block emerged. However, it was not supported by the full-reduced model comparison (Supplementary Table 4).

#### Time spent manipulating objects

The test block and the object novelty significantly influenced the time offspring spent manipulating objects (Fig. 2b and Supplementary Table 5). Offspring spent proportionally more time manipulating objects as the experiment progressed and offspring aged (Fig. 3b), and manipulating novel objects compared to familiar objects (Fig. 3c).

These effects were robust to model specification (see sensitivity check for singleton families; Supplementary Fig. 3 and Supplementary Table 6). However, when families with only one chick were excluded, one significant interaction between family size and test block emerged, supported by the full-reduced model comparison (Supplementary Table 6). Nonetheless, compared to the effects of test block and novelty, this interaction had a wide confidence interval overlapping zero.

### Social experience

#### Proximity between an offspring and another individual (parent or sibling) near the playground

**Fig. 4:**
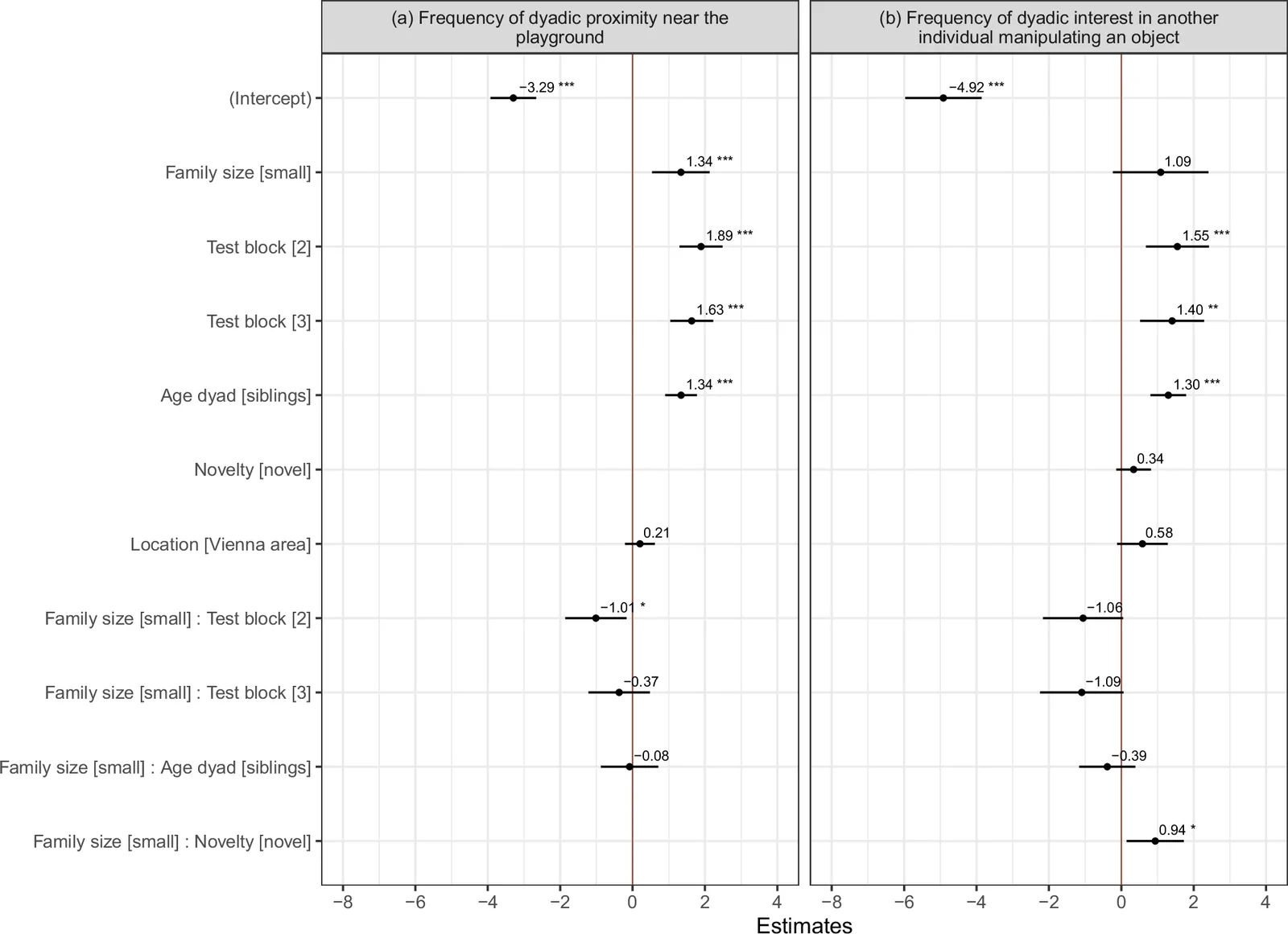
Model estimates of offspring proximity with other family members and interest in others manipulating objects near the playground. Estimates forest plots for the models investigating (**a**) the frequency of dyadic proximity between offspring and other family members (parent or sibling, initiated or received by an offspring) near the playground and **b** the frequency of dyadic interest by offspring in another individual manipulating an object (parent or sibling, emitted by an offspring). Frequencies are computed per dyad and test block. Reference levels for the categorical variables are: family size (large), test block (1), dyad age (siblings), novelty (familiar), and location (Cumberland Wildpark). Points represent model estimates, and error bars represent 95% confidence intervals. Sample size: **a** *N* = 196 observations, data aggregated per dyad (*n* = 69) and test block (*n* = 3), 11 values missing due to two families with missing data in test block one; **b** *N* = 392 observations, data aggregated per dyad, test block and object novelty (*n* = 2); doubling the number of observations compared to (**a**). See Supplementary Tables 7 and 9 for the full model outputs. ***<0.001, **<0.01, *≤0.05.

**Fig. 5:**
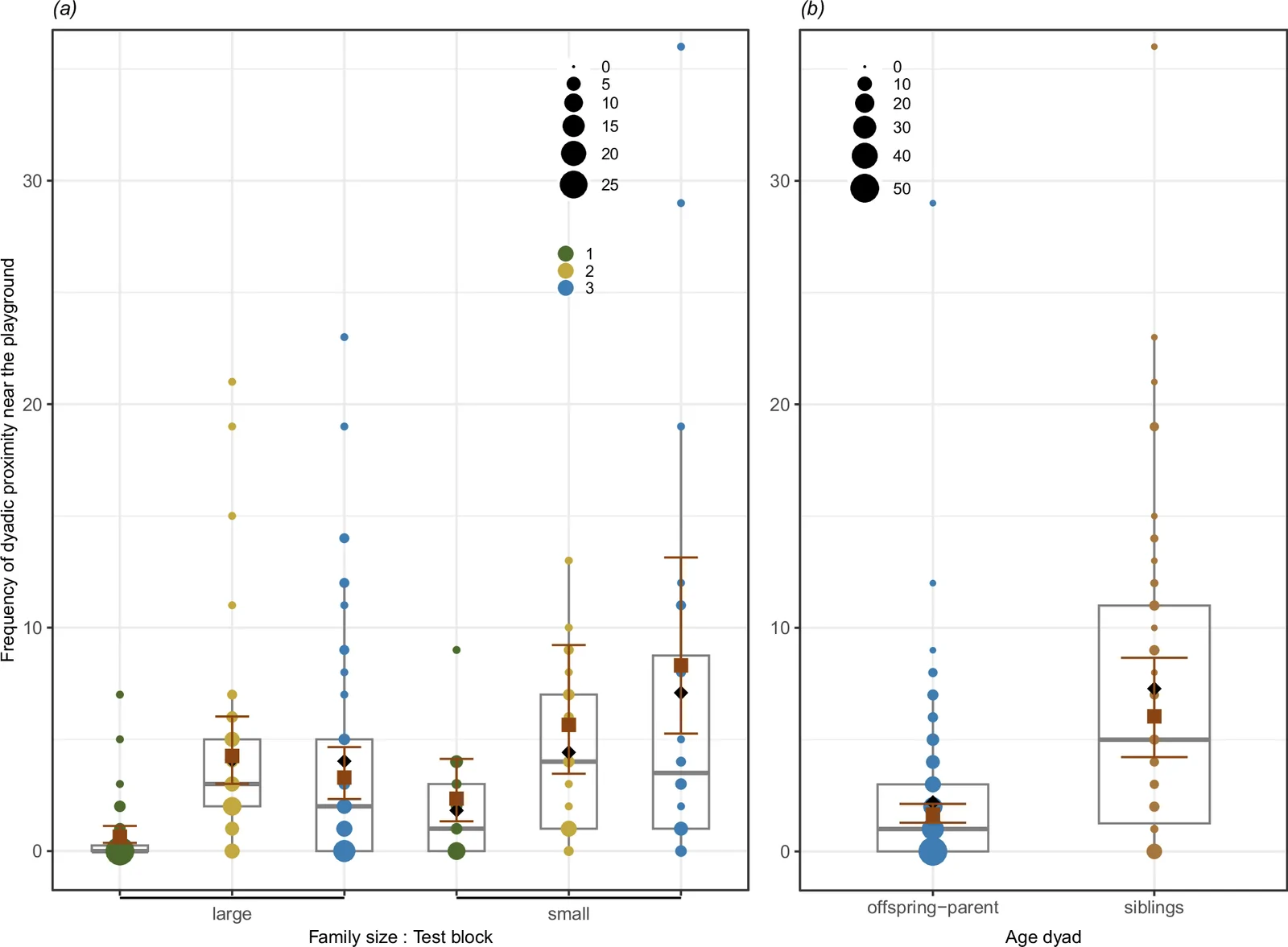
Raw data and model predictions of offspring proximity with other family members across family size, test block and dyad type. Raw data and model predictions for the frequency of dyadic proximity between offspring and other family members (parent or sibling, initiated or received by an offspring) near the playground according to the family size in interaction with test block (**a**) and the age of the dyad (**b**). Frequencies are computed per dyad and test block. Plots display raw data (dot size proportional to the number of observations) alongside: boxplots (showing the median and interquartile range), raw data means (black diamonds), and model predictions (brown square, estimated marginal means generated with the predict_response function, ggeffects R package) with 95% confidence intervals (error bars). The plots were generated using ggplot2.

The family size, test block, age of the dyad and, to a lesser extent, the interaction between family size and test block significantly influenced the frequency of proximity between offspring and other family members (parent or sibling) around the playground (initiated or received by an offspring) (Fig. 4a and Supplementary Table 7). Offspring in small families generally showed more frequent proximity around the playground than in large families (Fig. 5a). However, this effect was mediated by the test block. In particular, in the first test session, shortly after fledging, large family offspring showed a particularly low proximity rate compared to offspring from small families (Fig. 5a). Siblings were more frequently seen in proximity around the playground than offspring with their parents (Fig. 5b).

These effects were robust to model specification, all model results remaining consistent when single-chick families were excluded (see sensitivity check for singleton families; Supplementary Fig. 4 and Supplementary Table 8).

#### Interest by offspring in another individual (parent or sibling) manipulating an object

**Fig. 6:**
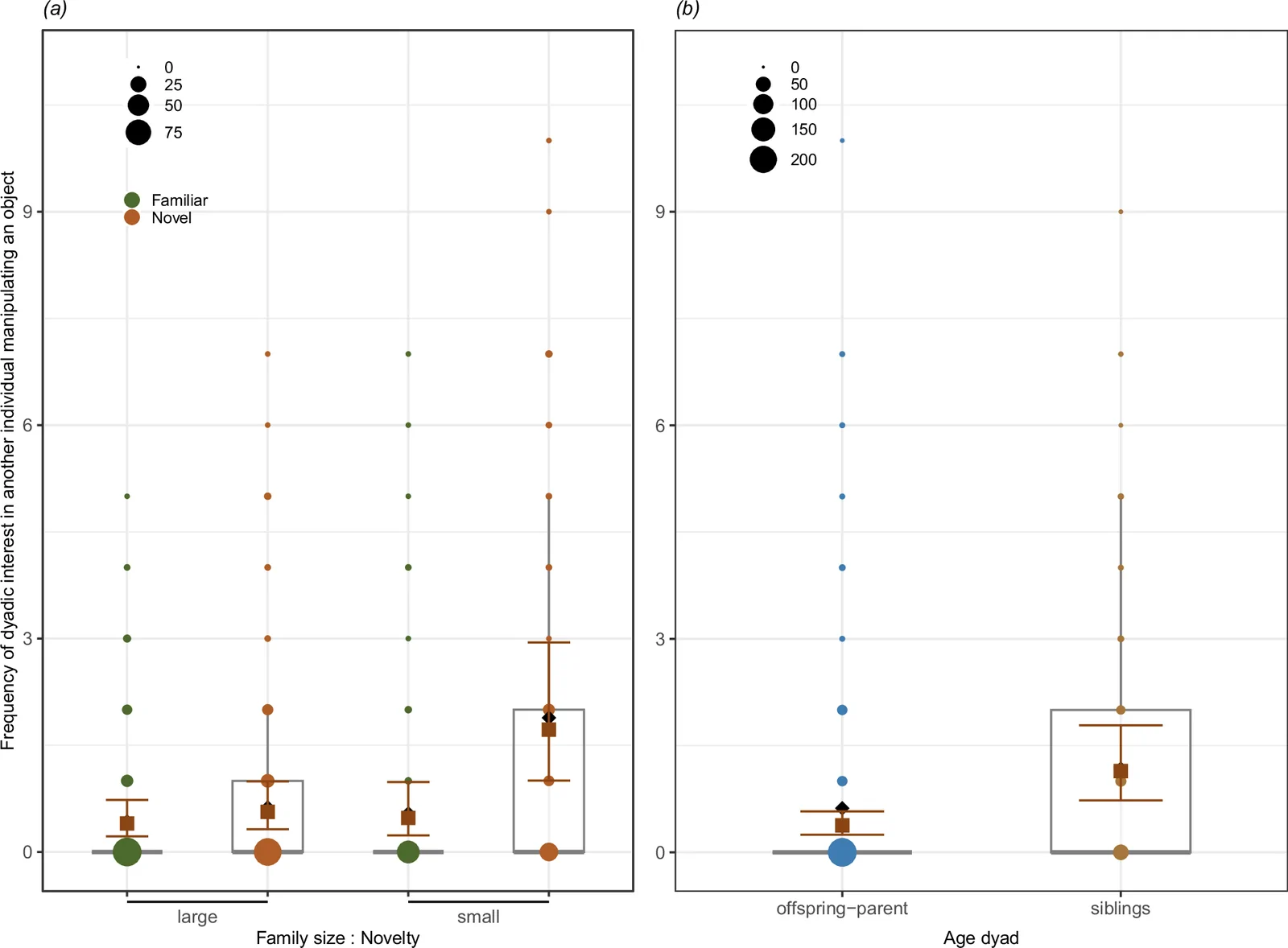
Raw data and model predictions of offspring interest in another individual manipulating an object across family size, object novelty and dyad type. Raw data and model predictions for the frequency of dyadic interest by offspring in another individual manipulating an object (parent or sibling, emitted by an offspring) according to family size in interaction with the object novelty (**a**) and the age of the dyad (offspring-parent, siblings) (**b**). Frequencies are computed per dyad and test block. Plots display raw data (dot size proportional to the number of observations) alongside: boxplots (showing the median and interquartile range), raw data means (black diamonds), and model predictions (brown square, estimated marginal means generated with the predict_response function, ggeffects R package) with 95% confidence intervals (error bars), generated using ggplot2. To better visualise the patterns, we adjusted the limits with coord_cartesian function, which allows changing the display without excluding any data ((**a**) one point outside the visual range: small, novel, interest frequency = 26; (**b**) one point outside the visual range: siblings, interest frequency = 26).

The test block, the age of the dyad and the interaction between family size and object novelty significantly influenced the likelihood of offspring ‘showing interest’ in another individual (parent or sibling) (Fig. 4b and Supplementary Table 9). Regardless of family size, offspring showed greater interest in others as the experiment progressed and they aged (Fig. 4b). Depending on family size, offspring from small families generally showed more interest in others than those from large families, but they were more likely to show interest when another individual manipulated a novel object than a familiar one, while large family offspring showed no preference (Fig. 6a). Across all dyads, siblings were more often the focus of interest of their peers than parents (Fig. 6b).

These effects were robust to model specification (see sensitivity check for singleton families; Supplementary Fig. 5 and Supplementary Table 10). However, when single-chick families were excluded, the interaction between family size and test block became significant, and was supported by the full-reduced model comparison (Supplementary Fig. 5 and Supplementary Table 10). This suggests that family composition may influence the expression of this interaction. Since all other effects remained stable and this result was observed on a subset of data, it should be interpreted with caution.

### Descriptive analyses

#### Order of access

**Fig. 7:**
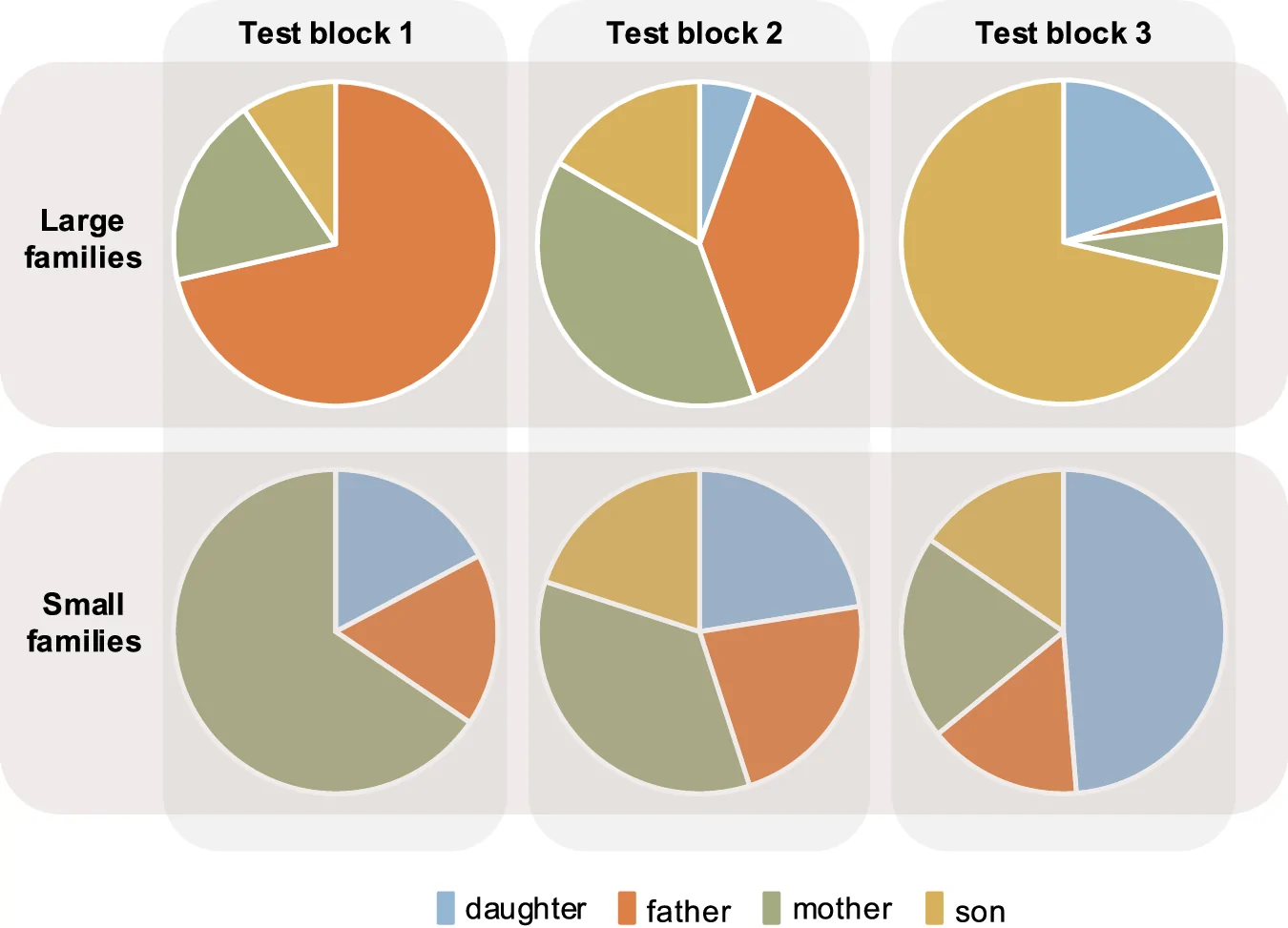
Relative percentages of access priority to the playground across family size, test block and individual identity. Pie chart showing the relative percentages of access priority to the playground (i.e. first to access), according to the family size, test block, and individual identity (daughter, mother, father, son). The percentages are computed over the total number of first-access events per family size and test block (large families: 21, 36, 35; small families: 29, 40, 39). Note that some families, particularly small ones, consisted of single-sex broods; therefore, not all individual identities could be represented in every case.

Over 246 sessions, we recorded 200 instances of an individual arriving first at the playground (Fig. 7). For 39 sessions, no individuals accessed the playground (19 in the first test block, 10 in test block two and 10 in test block three), and for seven sessions, we could not reliably identify the first individual. Over time, offspring increasingly arrived first at the playground compared to parents, irrespective of the family size. While parents were more likely to be first in test block one, offspring dominated first access by test block three. Interestingly, the dynamic differs for all sexes and depends on family size. Overall, males (sons and fathers) are favoured in large families, whereas in small families, females (daughters and mothers) are more likely to reach the playground first (Fig. 7). Thus, in large families, sons progressively gain priority, eventually occupying a significant share of first arrivals by test block three. In contrast, the trend is reversed in small families, where daughters more frequently arrive first than sons.

#### Playground usage

The frequencies of agonistic interactions, object removal from the boards, and transport away from the playground were similar across family sizes (Supplementary Fig. 6a–c). Adults were more often observed removing objects (Supplementary Fig. 6b), whereas offspring were slightly more often observed transporting them away (Supplementary Fig. 6c). Not all family members accessed the playground or manipulated an object or a board during every test session. Among those who did, a slightly larger share of offspring appeared to access the playground than adults (Supplementary Fig. 6d), and a slightly larger share of small-family offspring appeared to manipulate the playground (i.e. an object or a board) per session than offspring from large families or adults (Supplementary Fig. 6e).

Finally, if offspring, particularly in small families, appeared to spend slightly more time near the playground or manipulating objects than adults did, these differences were again associated with large, strongly overlapping standard deviations (Supplementary Figs. 7 and 8). In addition, all family members, irrespective of family size, appeared to spend similar time standing on a board (Supplementary Figs. 9), and individuals’ mean bout duration of engagement with the playground (i.e. time spent near the playground, manipulating objects or standing on a board) did not appear to be affected by family size or age class (Supplementary Figs. 7–9).

## Discussion

We investigated the development of object-directed behaviours in 25 juvenile ravens raised in families with varied numbers of offspring. Family size did not significantly affect offspring’s access to the playground setup nor the duration of object manipulation; yet, it influenced social patterns around objects, in the opposite direction as predicted. In addition, we found the predicted increase in object manipulation over time and a preference for novel objects. As object manipulations were not functional and occurred spontaneously and repeatedly in a relaxed atmosphere, they meet Burghardt’s criteria for play^35^.

Contrary to our initial expectations, our findings revealed that offspring from small families (one to two offspring) spent as much time near the playground and manipulating objects as the offspring from large families (three offspring). However, we found that small-family offspring engaged more frequently with their conspecifics around objects than those in large families. We had anticipated that the play context would foster mechanisms such as emotional contagion^36,42^. We, therefore, expected to observe more individual and social object play in large families due to the increased number of potential social partners. It could still be possible that emotional contagion had triggered play in the juvenile ravens, but the differences in family size were too subtle to affect its degree (i.e. for a young raven, it did not matter whether one or two siblings were playing).

Another explanation could be that our setup, consisting of two large boards with objects, might have encouraged monopolisation by some individuals, which could have resulted in increased competition for access to (objects at) the playground in large families as compared to small families. We recently described similar family size effects in the context of foraging^49^ (see also ref. ^50^). Aligned with the assumption of restricted access, we found that females (mothers and daughters) gained earlier access to the playground in small families, while males (fathers and sons)—who are typically dominant over females in the hierarchy and often more competitive than females^28,46,51^—were the first to access objects in large families. However, this pattern might have been reinforced by the fact that many of our small families consisted of female single-sex broods (four single-sex families out of six, three being composed of females), potentially creating a unique social environment that favoured females, unlike large, all mixed-sex families (Supplementary Table 1)^49^. Similar arguments have been brought forward to explain the amount of play in sex-biased and/or small litters of kittens^52,53^.

That more siblings mean fewer resources for the individual and hence increased competition is a common phenomenon in humans and non-human animals, and forms the basis of the so-called developmental stress hypothesis^54^. To our knowledge, only a few studies have addressed this hypothesis, or related concepts, in the play context. For instance, a recent study found birth order to affect toy choice in human children^55^. To our surprise, we found little support for the hypothesis’ key assumption—that offspring number leads to increased competition for resources—in our current study. Even though the offspring of differently sized families varied in gaining first access to the playground (see above), they all spent similar amounts of time at the playground (i.e. models analysing time spent in zone 1 and manipulating objects), indicating that an increase in family size did not prevent individuals from accessing the setup. Our modelling results are further supported by descriptive data on playground usage (Supplementary Figs. 6–10): Firstly, the aggression rate at the boards with objects was low and not affected by family size (Supplementary Fig. 6a). While low rates of aggression might be attributed to the young age of the offspring, another study conducted on the same families revealed pronounced rates of aggression already at that stage of development, when access to food was manipulated^49^. Secondly, the likelihood of removing objects from the boards and transporting them away from the playground was similar across family sizes (Supplementary Fig. 6b, c). Hence, we do not have any indication that birds from large families tried to avoid competition at the playground by removing items more often than birds from small families. Thirdly, we did not observe any patterns indicative for crowding effects, such as many short visits to a board, which could indicate frequent interruptions and increased competition for space, in large families as compared to small families (Supplementary Fig. 7). Finally, not all family members participated in the playground during every test session (Supplementary Fig. 6d, e), but all offspring engaged with the playground at least once (Supplementary Fig. 10). Altogether, these results speak against the assumption that ravens in large families faced increased competition over objects due to spatial limitations at the playground. Accordingly, we consider differences in competition of limited importance for explaining our key findings with respect to family size, i.e. that small-family offspring showed more frequent proximity to other family members and greater interest in others, especially when manipulating novel objects.

A plausible alternative explanation for our results could be that family size affected the quality of social relationships and the distribution of attention between siblings. On one hand, offspring of small families might be more attracted to each other, eventually forming closer bonds, than offspring of larger families^56^, which could have affected their presence pattern in the experiment. On the other hand, the offspring’s attention to social partners might become more diluted in large families than in small families, especially since siblings, rather than parents, are the focus of attention of young ravens (here, in terms of proximity and interest in others’ manipulations)^46^. The tolerance/bonding argument particularly fits the results of the first test block, when offspring from small and large families differed in their proximity at the playground. This block occurred early in development, when the establishment of relationships was in progress and possibly easier to achieve with fewer than with more family members^46^. Supporting the idea of a dilution of attention in large families, small-family offspring were not only selective about whom to watch (siblings over parents in all families) but also in what to watch, showing greater interest in others when manipulating novel objects. Such a social preference was absent in large families. This pattern aligns with findings from a previous study on social attention, conducted when our offspring had become independent from their families and were experienced with same-age peers originating from different families^56^. In this playback study, offspring from small families were more selective in their response to conspecific contact calls, paying longer attention to calls from familiar non-kin than offspring from large families^56^. We can thus speculate that offspring from small families value social information more highly, and consequently have more attentional space and motivation to engage in social interactions, than offspring from large families. In contrast, offspring from large families have to divide their attention and social investment among siblings, resulting in a more diffuse focus on individual social partners than in offspring from small families. Having one or more siblings may thus lead to different competencies.

Our study confirmed that as the juveniles matured, they increasingly spent time near and interacting with the playground, as well as with their conspecifics. This increase in object play^37,38^ combined with the juveniles’ attraction to novelty^39^ is thought to serve important developmental functions, supporting the refinement of motor skills necessary for prey capture and aiding the critical transition from parental provisioning to independent feeding^57^. Likewise, increased responses to and interactions with siblings over the first months post-fledge corroborate previous findings on ravens’ social learning^41^, caching^43^, gaze following^45^ and the formation of social coalitions^46^. Overall, these results highlight the importance of this early life period for developing socio-cognitive skills in this species and support the assumption that social competence starts developing in the family phase before juveniles enter socially dynamic non-breeder groups.

Our findings provide hints that the offspring’s sex composition within families might be an additional factor to explain variation in object play, such as first access to objects at the playground; possibly, it also affects the type of play and quality of interactions between siblings. The latter is well known from studies on human children, as boys and girls exhibit different toy preferences^58^ and play styles^59^, preferring playmates of their own gender group. Comparable studies on non-human animals, however, remain equivocal: for instance, female-only litters showed increased social play in mice^60^, whereas no such effect was found in kittens^52^.

Other factors that might explain (parts of) the observed variation in raven play concern individual differences between siblings and differences in the parenting styles of the adults. Indeed, ravens are renowned for exhibiting large individual variation in object-oriented behaviour during exploration^61,62^, which might extend to the context of object play. Likewise, breeders have been reported to treat their fledged offspring differently^63^. In our current study, we statistically accounted for individual identity, dyad identity and family identity, respectively. Future studies on play could be designed to address these factors specifically.

In Western Australian magpies (*Cracticus tibicen dorsalis*), group size correlates positively with the birds’ performance in cognitive tasks^64,65^. Although little is known about magpies’ object play, it has recently been shown that social interactions among group members (notably the amount of aggression received) shape the performance of juveniles in these tasks^65^. Note that, as Australian magpies have only very few young per group per year, older group members rather than siblings are likely responsible for the observed pattern^65^. Still, our findings may hint towards similar mechanisms, with group size affecting the patterns of interaction between family members and ultimately juveniles’ learning and development opportunities^66,67^.

Taken together, our results highlight the value of studying subtle, naturally occurring brood sizes in parent-raised individuals when studying developmental behaviours. In this study, ravens were parent-raised in naturally sized family groups, unlike earlier ontogeny studies in this species (cited above, where birds were hand-raised by human foster parents and housed in peer groups of same-aged individuals). They were raised in families ranging from one to three offspring per pair, thus having a narrower brood range compared to the species’ reproductive potential (one–seven eggs), but aligning with the most commonly observed brood sizes in both wild and captive populations^31,34^. Still, we show that even small variations in brood size can have measurable effects on developmental experiences. Family size significantly influenced how frequently offspring were in proximity with others, showed interest in their activities and were selective in what to watch. This supports the hypothesis that family size plays a subtle but pivotal role in shaping the early social environment and its consequences on the development of social and exploratory behaviours. Future studies should continue to investigate other social parameters during upbringing, such as the offspring sex ratio, parenting style, or more generally, interactions between parents and offspring, as well as among siblings.

Our set of small families included two families with a single offspring (others had two offspring). As a sensitivity check, we therefore ran all models excluding single-offspring families to verify the robustness of the patterns to this particular family composition. The main results remained consistent across different model specifications, indicating that singleton families did not influence these observed effects. However, in all but one model, the interaction between family size and test block appeared to be sensitive to the inclusion of single-offspring families. Due to the sensitivity of this interaction’s expression to sampling variation and the structural limitations of the dataset, we will not interpret it further here. Nonetheless, this emphasises the need to compile even larger datasets (especially for single-offspring families, which produce proportionally fewer statistical subjects than larger ones) to perform analyses directly using the number of chicks rather than aggregated size factors, in order to understand how slight variations in the number of partners can influence social dynamics and experiences within the rearing unit.

In conclusion, our results indicate that natural variation in family size influences social attention and interactions around objects. These findings highlight that even subtle, naturally occurring variations in early social environments can shape developmental trajectories. We hope our study inspires further research into the proximate mechanisms driving the development of social competencies in early life, across diverse social systems and species. Such investigations could deepen our understanding of how early-life experiences shape social skills, offering valuable insights into social behaviour’s evolutionary and ecological underpinnings.

## Supplementary information

Supplementary Information

Reporting summary

Supplementary Code 1

Supplementary Data 1

## Data Availability

The datasets used in this study for all statistical analyses and figures are available as part of the electronic supplementary material (Supplementary Data 1).

## Code availability

The R code used in this study to run all statistical analyses and process figures is available as part of the electronic supplementary material (Supplementary Code 1).
