# Supplementary material for: Subtle differences in family size mediate object play in young ravens (Corvus corax)
Source: Commun Biol. 2026 Jul 11;9:1460. doi: 10.1038/s42003-026-10290-0 (PMC13737195; doi:10.1038/s42003-026-10290-0)
Supplement: Supplementary file 1 [file 42003_2026_10290_MOESM1_ESM.pdf]

SUPPLEMENTARY MATERIALS

Subtle differences in family size mediate object play in young ravens (*Corvus corax)*

Authors: Palmyre H. Boucherie[1] and Valentine Comin[1,2 *], Thomas Bugnyar[1,2,3]*

1. *Department of Behavioural and Cognitive Biology, University of Vienna, Djerassiplatz 1, 1030 Vienna, Austria*
2. *Haidlhof Research Station, University of Vienna and University of Veterinary Medicine Vienna, 2540 Bad Vöslau, Austria*
3. *Konrad Lorenz Research Center, University of Vienna, Fischerau 11, A-4645 Grünau, Austria*

** Co-first authors*

Corresponding author: Palmyre H. Boucherie, palmyre.boucherie@gmail.com

Supplementary Table 1. Characteristics of all families involved in the experiment between 2019 and 2021. Note that one offspring (from Rocky-Joey 2020) has unknown sex (indicated by '?') and was excluded from the first two models in the analyses (including individual's sex in the predictors). *For George-Nobel, the eggs were replaced by eggs of another pair of our colony, not included in this study. For locations, abbreviations represent: Cumberland Wildpark (C.W.), Haidlhof Research Station (H.R.S.), Schönbrunn Zoo Vienna (S.Z.V.).

Year	Family	Breeding pair	Number of chicks and sex ratio [male,female]	Family size	Location	Number of test sessions
2019	AM19	Arthus-Martha	2 [0,2]	F-	C.W.	24
2019	GN19	George-Nobel	2* [0,2]	F-	H.R.S.	24
2019	ML19	Matte-Lellan	3 [1,2]	F+	H.R.S.	24
2019	RJ19	Rocky-Joey	3 [2,1]	F+	H.R.S.	24
2019	RM19	Rufus-Munia	1 [1,0]	F-	S.Z.V.	22
2019	TH19	Tom-Heidi	3 [2,1]	F+	C.W.	24
2020	AM20	Arthus-Martha	3 [2,1]	F+	C.W.	16
2020	GN20	George-Nobel	2* [1,1]	F-	H.R.S.	24
2020	RJ20	Rocky-Joey	2 [1,?]	F-	H.R.S.	24
2020	TH20	Tom-Heidi	1 [0,1]	F-	C.W.	16
2021	RM21	Rufus-Munia	3 [2,1]	F+	S.Z.V.	24

Supplementary Note 1. Details about the equipment used for data collection.

At Haidlhof, Panasonic HC-V808 and Canon cameras and a tripod Velbon Videomate 438 were used; in 2020 and 2021, dome cameras installed on top of the aviary were used for the global view on the aviary. At the Konrad Lorenz Research Center, Panasonic HC-V808, Sony cameras and, on some occasions, also a Canon and GoPro cameras were used. At Zoo Vienna, two Panasonic V808 cameras, a JVC Everio GZ-HM30BU camera, a tripod ALPHA Design ALPHA 1000 and a tripod Velbon Videomate 438 were used.

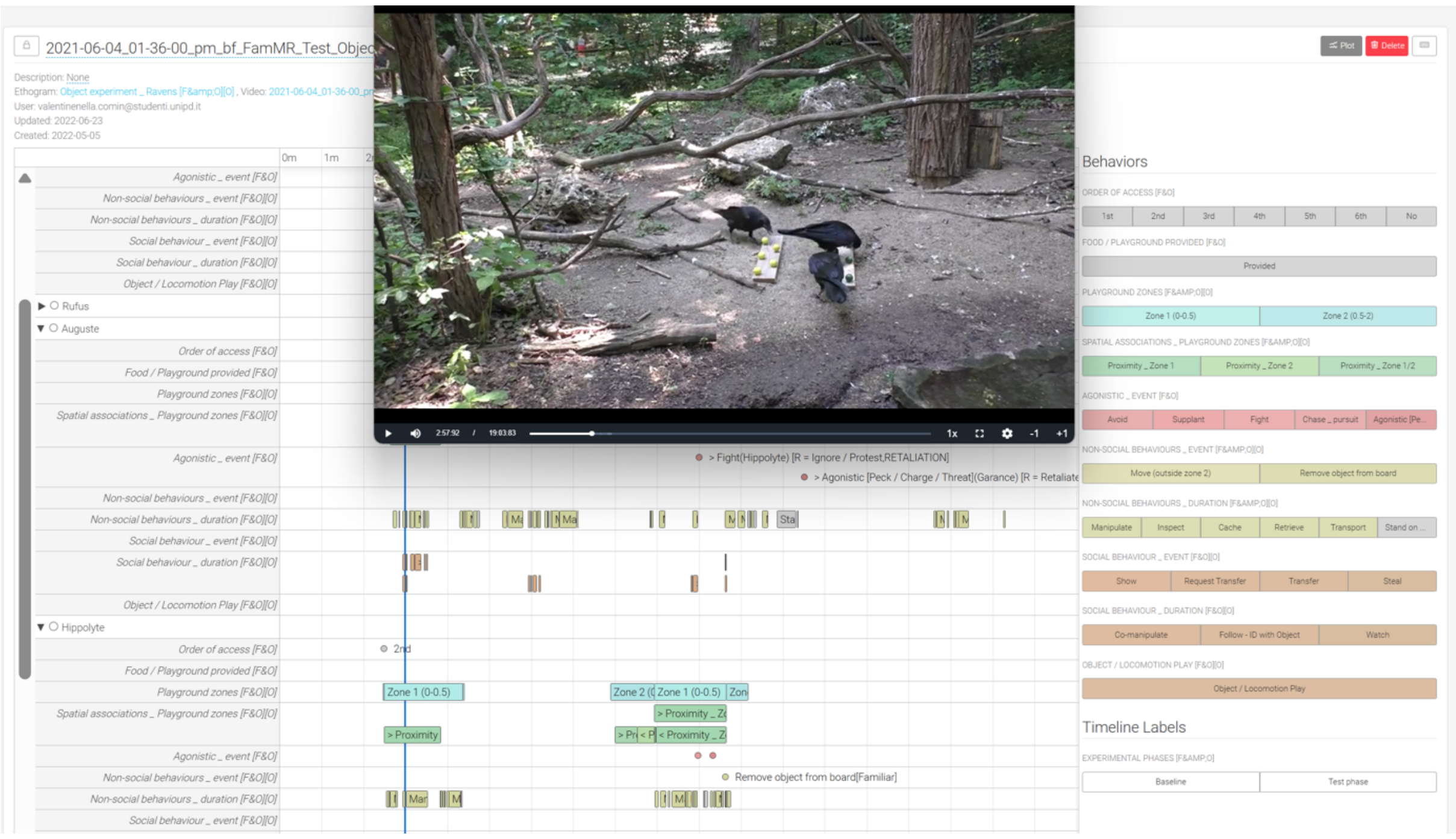

Supplementary Figure 1. Screenshot of the coding platform on Loopy coding software for quantitative video analysis from Loopbio (http://loopb.io, loopbio gmbh, Vienna, Austria). The screenshot displays the movie frame (movable), a section of the ethogram (on the right), and the scoring window in the centre, with data aggregated per focal individual and coded behaviours or interactions. Duration bouts are shown as rectangles, and events as dots.

Supplementary Table 2. Behavioural ethogram.

Behavioural category	***Behaviour***	***Description***
	**Non-social behaviours**	
**Order of access**	*1st*	Ranking order in which individuals enter zone 1 for the first time during each test session. Only the identity of the first individual to approach the playground was analysed.

**Zone 1**	*Zone 1*	The individual is within a radius of 0-0.5m around the playground, or one of the two boards composing it.
**Object manipulation**	*Manipulate*	An individual interacts with an item from the playground (movable object, non-movable object or the board itself) using its beak or foot.
	Transport	The individual flies or walks while carrying an object in its beak.
	Cache	The individual hides an object by covering it with various materials such as twigs, leaves and moss.
	Retrieve	One bird retrieves an object that was previously cached.
		**Social behaviours**
**Show interest**	*Watch*	One bird is in proximity with another one and observes it handling an object without any attempt to get it.
	Follow	One bird walks or flies in the same direction as another individual who is carrying an object and with whom it was previously in proximity.
**Proximity**	*Proximity in zone 1*	Two individuals are 0-1m away from each other and within 0.5m of the playground or of the two boards composing it.

Supplementary Table 3. GLMM results for the model investigating the proportion of time spent near the object playground over the total observation time (within zone 1), computed per offspring and test block. Reference levels are family size (large), test block (1), sex (female), and location (Cumberland Wildpark). For each interaction term, we report the overall significance by comparison with the full model comprising the full set of predictors to a reduced

model lacking the considered interaction: -{interaction term}. Comparisons were performed using a likelihood ratio test, from which we report the AIC, $\chi 2$, degree of freedom and p-value. Significant p-values are marked in bold. *** < 0.001, ** < 0.01, * ≤ 0.05.

	**Proportion of time spent around the object playground**				
Predictors	*Estimates*	*std. Error*	*CI*	*Statistic*	*p*
(Intercept)	-3.75	0.33	-4.40 – -3.09	-11.26	**<0.001**
Family size [small]	0.64	0.45	-0.25 – 1.52	1.42	0.157
Test block [2]	1.34	0.28	0.80 – 1.89	4.86	**<0.001**
Test block [3]	1.43	0.27	0.89 – 1.97	5.22	**<0.001**
Sex [male]	0.17	0.28	-0.37 – 0.71	0.60	0.546
Location [Vienna area]	0.41	0.23	-0.03 – 0.85	1.82	0.069
Family size [small] × Test block [2]	-0.40	0.42	-1.23 – 0.42	-0.96	0.337
Family size [small] × Test block [3]	-0.03	0.41	-0.84 – 0.77	-0.08	0.938
Family size [small] × Sex [male]	-0.44	0.45	-1.33 – 0.45	-0.97	0.333
**Random Effects**					
σ^2	0.24				
$\tau_{00\ ID}$	0.14				
ICC	0.37				
N_{ID}	24				
Observations	68				
Marginal R^2 / Conditional R^2	0.529 / 0.701				
AIC	-199.3				

Full-reduced model comparisons

**AIC Null model**	-168.3, Chisq = 47.026, Df =8, p-value **<0.001**
AIC Full - {Family size : Test block}	-201.6, Chisq = 1.772, Df =2, p-value = 0.41
AIC Full - {Family size : Sex}	-200.4, Chisq = 0.922, Df =1, p-value = 0.34

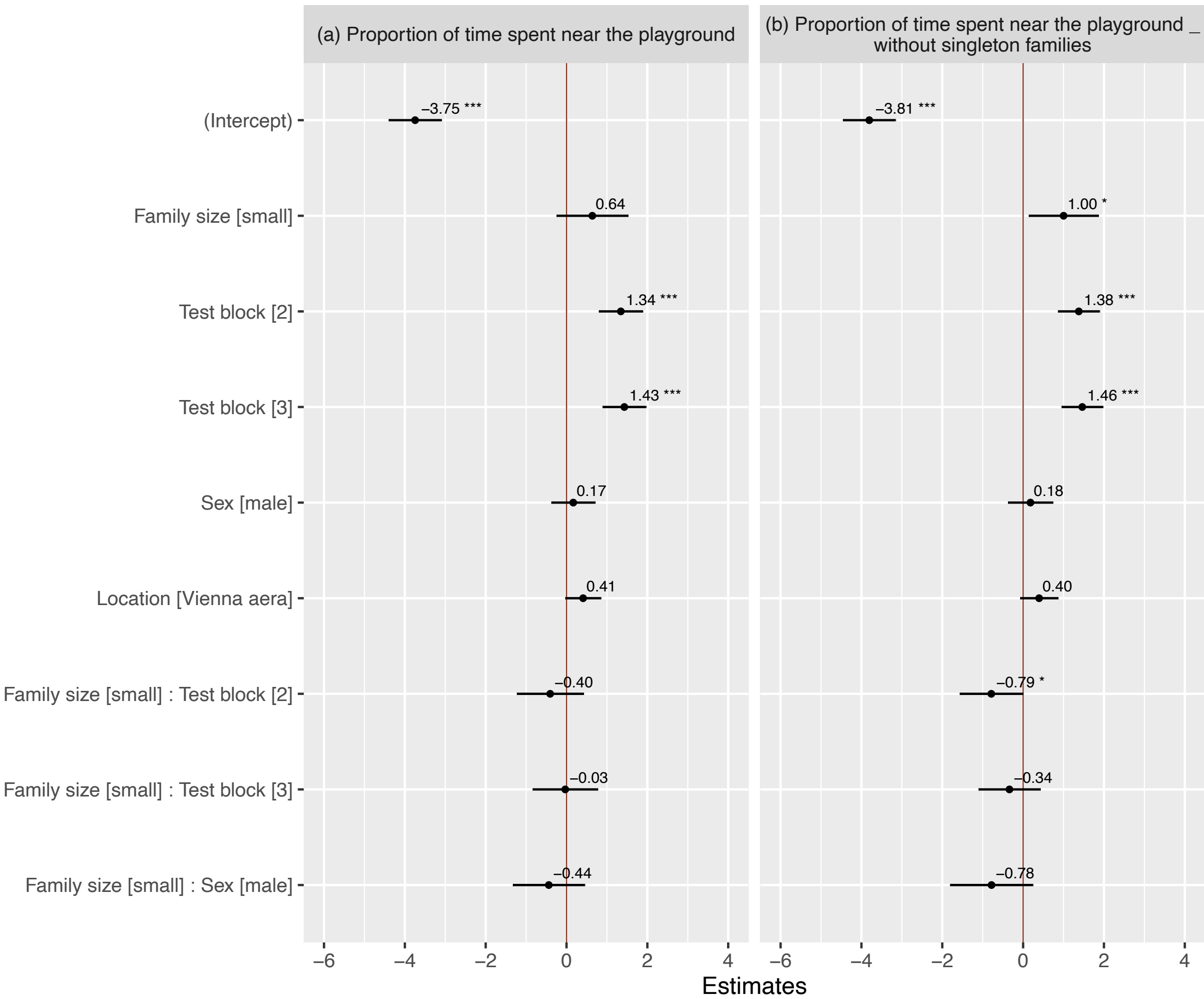

Supplementary Figure 2. Sensitivity check: comparison of estimates forest plots for the models investigating the proportion of time spent near the playground (i.e., within a 50-cm zone around the playground) (a) with and (b) without singleton families (with a single chick), computed per offspring and test block. Reference levels for the categorical variables are: family size (large), test block (1), sex (female), novelty (familiar), and Location (Cumberland

Wildpark). See electronic supplementary material, Tables S3 and S4 for the full model outputs. *** < 0.001, ** < 0.01, * ≤ 0.05.

Supplementary Table 4. Sensitivity check: GLMM results for the model investigating the proportion of time spent near the object playground over the total observation time (within zone 1), without singleton families (with a single chick), computed per offspring and test block. Reference levels are family size (large), test block (1), sex (female), and location (Cumberland Wildpark). For each interaction term, we report the overall significance by comparison with the full model comprising the full set of predictors to a reduced model lacking the considered interaction: -{interaction term}. Comparisons were performed using a likelihood ratio test, from which we report the AIC, χ 2, degree of freedom and p-value. Significant p-values are marked in bold. *** < 0.001, ** < 0.01, * ≤ 0.05.

	**Proportion of time spent around the object playground _ without singleton families**				
Predictors	*Estimates*	*std. Error*	*CI*	*Statistic*	*p*
(Intercept)	-3.81	0.33	-4.46 – -3.16	-11.49	**<0.001**
Family size [small]	1.00	0.44	0.14 – 1.86	2.28	**0.023**
Test block [2]	1.38	0.26	0.86 – 1.89	5.24	**<0.001**
Test block [3]	1.46	0.26	0.95 – 1.97	5.62	**<0.001**
Sex [male]	0.18	0.28	-0.37 – 0.74	0.64	0.522
Location [Vienna area]	0.40	0.24	-0.07 – 0.86	1.65	0.098
Family size [small] × Test block [2]	-0.79	0.40	-1.57 – -0.01	-1.98	**0.048**
Family size [small] × Test block [3]	-0.34	0.39	-1.10 – 0.43	-0.87	0.385

Family size [small] × Sex [male]	-0.78	0.52	-1.81 – 0.24	-1.50	0.135
**Random Effects**					
σ^2	0.22				
$\tau_{00\ ID}$	0.17				
ICC	0.44				
N_{ID}	22				
Observations	63				
Marginal R^2 / Conditional R^2	0.524 / 0.735				
AIC	-191.4				
**Full-reduced model comparisons**					
**AIC Null model**	-161.4, Chisq = 45.932, Df =8, p-value **<0.001**				
AIC Full - {Family size : Test block}	-191.1, Chisq = 4.265, Df =2, p-value = 0.12				
AIC Full - {Family size : Sex}	-191.2, Chisq = 2.161, Df =1, p-value = 0.14				

Supplementary Table 5. GLMM results for the model investigating the proportion of time spent manipulating objects, computed per offspring and test block. Reference levels are family size (large), test block (1), sex (female), novelty (familiar) and location (Cumberland Wildpark). For each interaction term, we report the overall significance by comparison with the full model comprising the full set of predictors to a reduced model lacking the considered interaction: -{interaction term}. Comparisons were performed using a likelihood ratio test, from which we report the AIC, χ 2, degree of freedom and p-value. Significant p-values are marked in bold. *** < 0.001, ** < 0.01, * ≤ 0.05.

	**Proportion of time manipulating objects**

Predictors	*Estimates*	*std. Error*	*CI*	*Statistic*	*p*
(Intercept)	-5.19	0.30	-5.78 – -4.61	-17.44	**<0.001**
Family size [small]	0.38	0.39	-0.39 – 1.16	0.98	0.329
Test block [2]	1.02	0.19	0.64 – 1.39	5.33	**<0.001**
Test block [3]	1.23	0.19	0.87 – 1.60	6.64	**<0.001**
Sex [male]	0.08	0.17	-0.24 – 0.41	0.50	0.614
Novelty [novel]	0.48	0.12	0.24 – 0.72	3.91	**<0.001**
Location [Vienna area]	0.45	0.28	-0.09 – 0.99	1.62	0.106
Family size [small] × Test block [2]	-0.15	0.29	-0.72 – 0.41	-0.53	0.594
Family size [small] × Test block [3]	0.27	0.27	-0.27 – 0.81	0.99	0.324
Family size [small] × Sex [male]	-0.48	0.37	-1.20 – 0.25	-1.29	0.197
Family size [small] × Novelty [novel]	0.10	0.18	-0.26 – 0.46	0.56	0.579
**Random Effects**					
σ^2	0.25				
τ_{00} ID:Family_Year	0.03				
τ_{00} Family_Year	0.12				
ICC	0.38				
N ID	24				
N Family_Year	11				
Observations	136				
Marginal R^2 / Conditional R^2	0.518 / 0.703				
AIC	-773.958				

Full-reduced model comparisons

**AIC Null model**	-699.9, Chisq = 94.071, Df =10, p-value **<0.001**
AIC Full - {Family size : Test block}	-773.5, Chisq = 4.5, Df =2, p-value = 0.10

AIC Full - {Family size : Sex}	-774.4, Chisq = 1.56, Df =1, p-value = 0.21
AIC Full - {Family size : Novelty}	-775.6, Chisq = 0.31, Df =1, p-value = 0.58

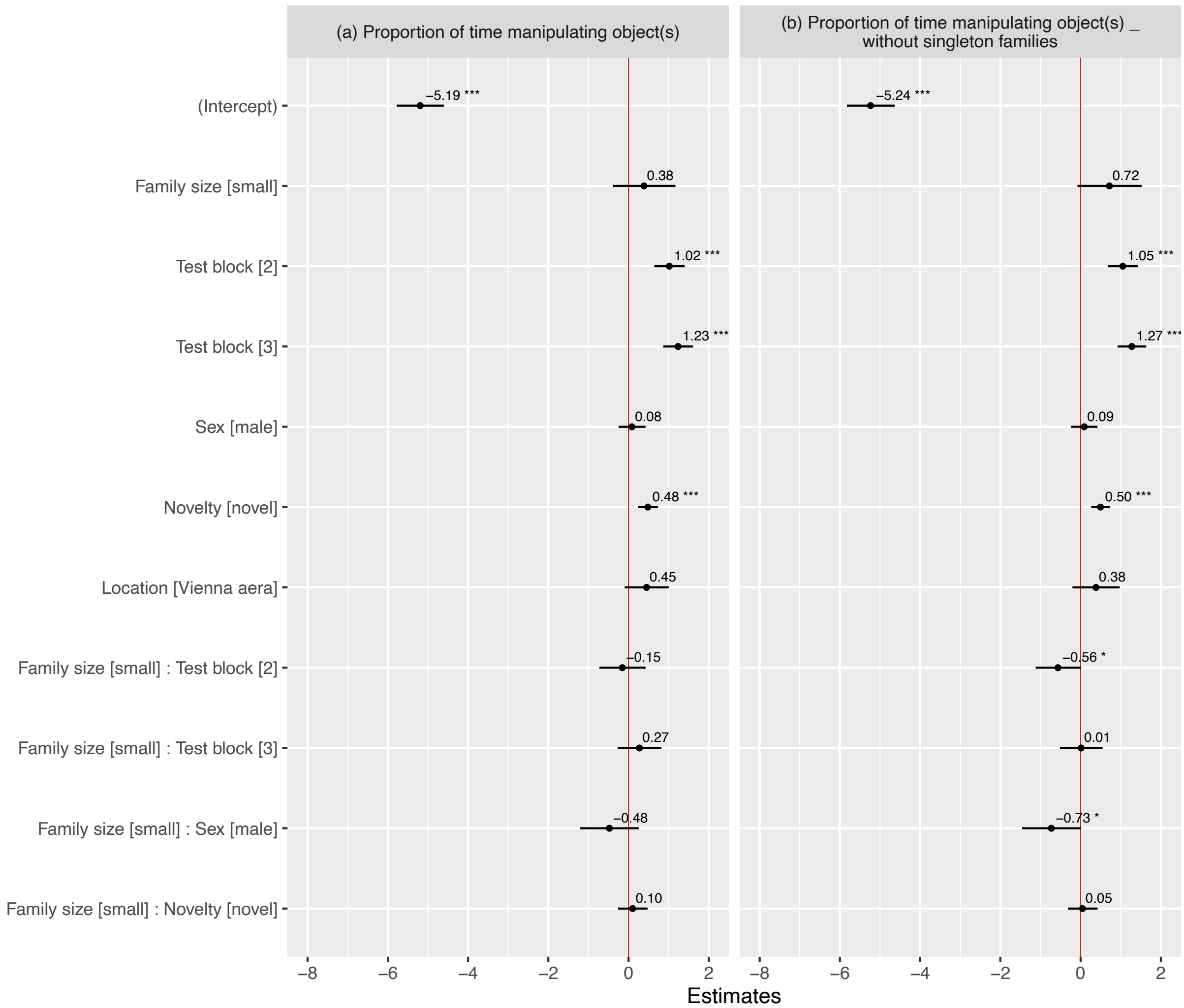

Supplementary Figure 3. Sensitivity check: comparison of estimates forest plots for the models investigating the proportion of time spent manipulating objects (a) with and (b) without singleton families (with a single chick), computed per offspring and test block. Reference levels for the categorical variables are: family size (large), test block (1), sex (female), novelty (familiar), and Location (Cumberland Wildpark). See electronic supplementary material, Tables S3 and S4 for the full model outputs. *** < 0.001, ** < 0.01, * ≤ 0.05.

Supplementary Table 6. Sensitivity check: GLMM results for the model investigating the proportion of time spent manipulating objects, without singleton families (with a single chick), computed per offspring and test block. Reference levels are family size (large), test block (1), sex (female), novelty (familiar) and location (Cumberland Wildpark). For each interaction term, we report the overall significance by comparison with the full model comprising the full set of predictors to a reduced model lacking the considered interaction: -{interaction term}. Comparisons were performed using a likelihood ratio test, from which we report the AIC, χ 2, degree of freedom and p-value. Significant p-values are marked in bold. *** < 0.001, ** < 0.01, * ≤ 0.05.

	**Proportion of time manipulating objects _ without singleton families**				
Predictors	*Estimates*	*std. Error*	*CI*	*Statistic*	*p*
(Intercept)	-5.24	0.30	-5.82 – -4.65	-17.47	**<0.001**
Family size [small]	0.72	0.41	-0.08 – 1.51	1.77	0.077
Test block [2]	1.05	0.18	0.69 – 1.41	5.72	**<0.001**
Test block [3]	1.27	0.18	0.92 – 1.62	7.12	**<0.001**
Sex [male]	0.09	0.16	-0.23 – 0.41	0.54	0.586
Novelty [novel]	0.50	0.12	0.27 – 0.72	4.26	**<0.001**
Location [Vienna area]	0.38	0.30	-0.20 – 0.97	1.28	0.199
Family size [small] × Test block [2]	-0.56	0.28	-1.12 – -0.01	-2.00	**0.046**
Family size [small] × Test block [3]	0.01	0.27	-0.51 – 0.54	0.04	0.967
Family size [small] × Sex [male]	-0.73	0.37	-1.45 – -0.00	-1.97	**0.049**
Family size [small] × Novelty [novel]	0.05	0.19	-0.32 – 0.41	0.25	0.801
**Random Effects**					
σ^2	0.24				

$\tau_{00\ \text{ID:Family\_Year}}$	0.04
$\tau_{00\ \text{Family\_Year}}$	0.13
ICC	0.42
N_{ID}	22
$N_{\text{Family\_Year}}$	9
Observations	126
Marginal R^2 / Conditional R^2	0.510 / 0.715
AIC	-736.573
**Full-reduced model comparisons**	
**AIC Null model**	-666.3, Chisq = 90.314, Df =10, p-value **<0.001**
**AIC Full - {Family size : Test block}**	-732.4, Chisq = 8.221, Df =2, p-value **< 0.05**
AIC Full - {Family size : Sex}	-735.3, Chisq = 3.314, Df =1, p-value = 0.07
AIC Full - {Family size : Novelty}	-738.5, Chisq = 0.063, Df =1, p-value = 0.80

Supplementary Table 7. GLMM results for the model investigating the frequency of dyadic proximity between offspring and other family members (adult or sibling, initiated or received by an offspring) near the playground, computed per dyad and test block. Reference levels are family size (large), test block (1), age dyad (offspring-parent) and location (Cumberland Wildpark). For each interaction term, we report the overall significance by comparison with the full model comprising the full set of predictors to a reduced model lacking the considered interaction: -{interaction term}. Comparisons were performed using a likelihood ratio test, from which we report the AIC, χ 2, degree of freedom and p-value. Significant p-values are marked in bold. *** < 0.001, ** < 0.01, * ≤ 0.05.

	**Frequency of dyadic proximity near the playground**				
Predictors	*Log-Mean*	*std. Error*	*CI*	*Statistic*	*p*

(Intercept)	-3.29	0.32	-3.92 – -2.66	-10.27	**<0.001**
Family size [small]	1.34	0.41	0.54 – 2.13	3.30	**0.001**
Test block [2]	1.89	0.30	1.30 – 2.49	6.23	**<0.001**
Test block [3]	1.63	0.30	1.04 – 2.23	5.40	**<0.001**
Age dyad [siblings]	1.34	0.22	0.90 – 1.78	5.97	**<0.001**
Location [Vienna area]	0.21	0.21	-0.21 – 0.62	0.98	0.327
Family size [small] × Test block [2]	-1.01	0.43	-1.86 – -0.17	-2.35	**0.019**
Family size [small] × Test block [3]	-0.37	0.43	-1.22 – 0.48	-0.85	0.393
Family size [small] × Age [siblings]	-0.08	0.40	-0.87 – 0.71	-0.20	0.840
**Random Effects**					
σ^2	1.32				
$\tau_{00\ Dyad:Family\_Year}$	0.11				
$\tau_{00\ Family\_Year}$	0.03				
ICC	0.09				
N_{Dyad}	69				
N_{Family_Year}	11				
Observations	196				
Marginal R^2 / Conditional R^2	0.393 / 0.449				
AIC	847.471				
**Full-reduced model comparisons**					
**AIC Null model**	925.6, Chisq = 94.082, Df =8, p-value **<0.001**				
**AIC Full - {Family size : Test block}**	849.9, Chisq = 6.442, Df =2, p-value < **0.05**				
AIC Full - {Family size : Age dyad}	845.5, Chisq = 0.041, Df =1, p-value = 0.84				

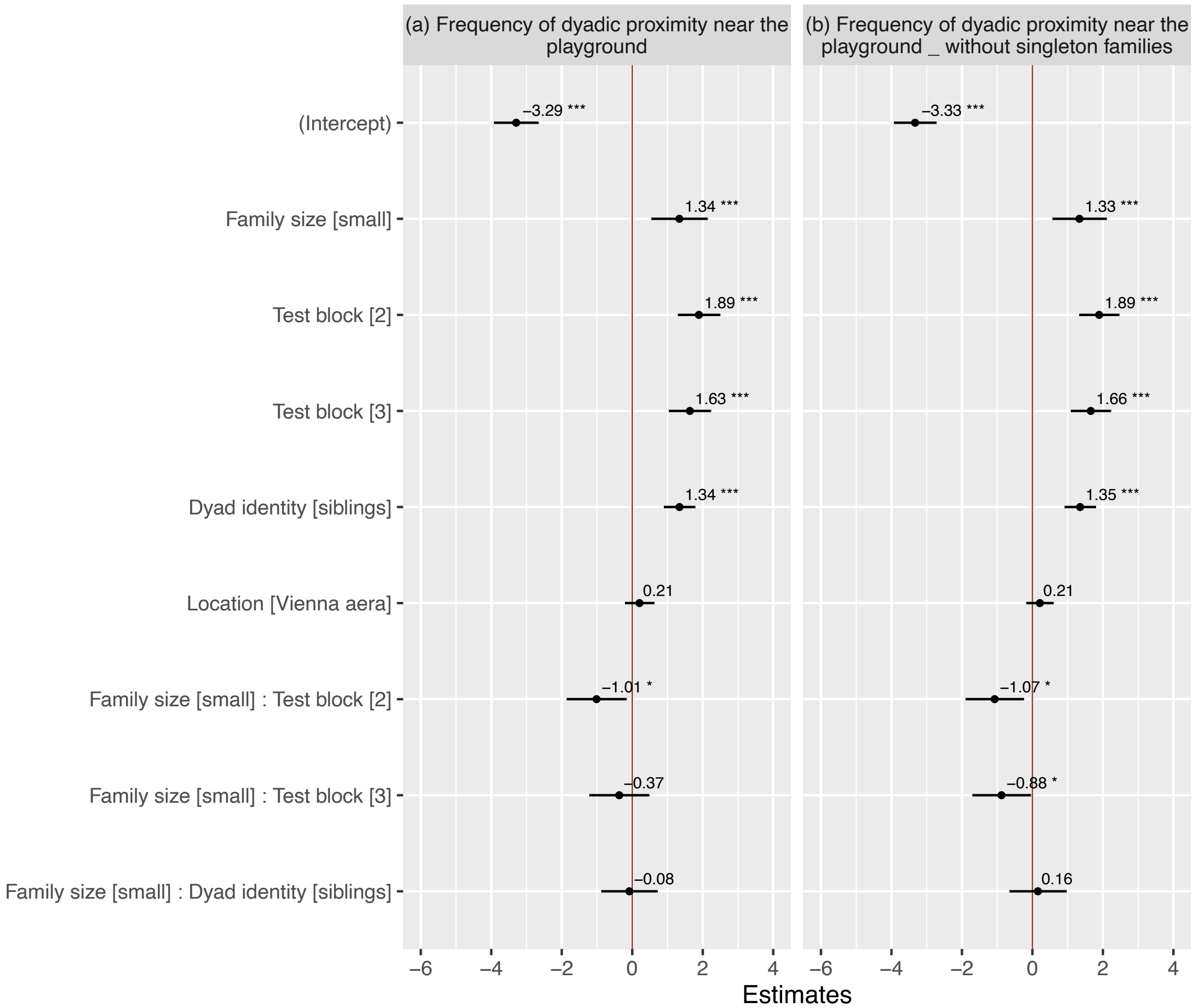

Supplementary Figure 4. Sensitivity check: comparison of estimates forest plots for the models investigating the frequency of dyadic proximity between offspring and other family members (adult or sibling, initiated or received by an offspring) near the playground (a) with and (b) without singleton families (with a single chick), computed per dyad and test block. Reference levels for the categorical variables are: family size (large), test block (1), age dyad (offspring-parent), novelty (familiar), and location (Cumberland Wildpark). See electronic supplementary material, Tables S5 and S6 for the full model outputs. *** < 0.001, ** < 0.01, * ≤ 0.05.

Supplementary Table 8. Sensitivity check: GLMM results for the model investigating the frequency of dyadic proximity between offspring and other family members (adult or sibling,

initiated or received by an offspring) near the playground, without singleton families (with a single chick), computed per dyad and test block. Reference levels are family size (large), test block (1), age dyad (offspring-parent) and location (Cumberland Wildpark). For each interaction term, we report the overall significance by comparison with the full model comprising the full set of predictors to a reduced model lacking the considered interaction: -{interaction term}. Comparisons were performed using a likelihood ratio test, from which we report the AIC, χ 2, degree of freedom and p-value. Significant p-values are marked in bold. *** < 0.001, ** < 0.01, * ≤ 0.05.

	**Frequency of dyadic proximity near the playground _ without singleton families**				
Predictors	*Log-Mean*	*std. Error*	*CI*	*Statistic*	*p*
(Intercept)	-3.33	0.30	-3.93 – -2.73	-10.92	**<0.001**
Family size [small]	1.33	0.39	0.57 – 2.10	3.41	**0.001**
Test block [2]	1.89	0.29	1.33 – 2.46	6.55	**<0.001**
Test block [3]	1.66	0.29	1.09 – 2.22	5.73	**<0.001**
Age dyad [siblings]	1.35	0.22	0.91 – 1.79	6.05	**<0.001**
Location [Vienna area]	0.21	0.19	-0.17 – 0.59	1.08	0.280
Family size [small] × Test block [2]	-1.07	0.42	-1.90 – -0.25	-2.55	**0.011**
Family size [small] × Test block [3]	-0.88	0.42	-1.70 – -0.05	-2.08	**0.037**
Family size [small] × Age dyad [siblings]	0.16	0.41	-0.65 – 0.96	0.38	0.705
**Random Effects**					
σ^2	1.33				
$\tau_{00\ Dyad:Family\_Year}$	0.16				
$\tau_{00\ Family\_Year}$	0.00				

ICC	0.11
N $_{Dyad}$	65
N $_{Family\_Year}$	9
Observations	186
Marginal R^2 / Conditional R^2	0.387 / 0.453
AIC	784.045
**Full-reduced model comparisons**	
**AIC Null model**	860.3, Chisq = 92.272, Df =8, p-value **<0.001**
**AIC Full - {Family size : Test block}**	786.8, Chisq = 6.738, Df =2, p-value **< 0.05**
AIC Full - {Family size : Age dyad}	782.2, Chisq = 0.144, Df =1, p-value = 0.70

Supplementary Table 9. GLMM results for the model investigating the frequency of dyadic interest by offspring in another individual manipulating an object (adult or sibling, emitted by an offspring), computed per dyad and test block. Reference levels are family size (large), test block (1), age dyad (offspring-parent), novelty (familiar) and location (Cumberland Wildpark). For each interaction term, we report the overall significance by comparison with the full model comprising the full set of predictors to a reduced model lacking the considered interaction: -{interaction term}. Comparisons were performed using a likelihood ratio test, from which we report the AIC, χ 2, degree of freedom and p-value. Significant p-values are marked in bold. *** < 0.001, ** < 0.01, * ≤ 0.05.

	**Frequency of dyadic interest in another individual manipulating an object**				
Predictors	*Log-Mean*	*std. Error*	*CI*	*Statistic*	*p*
(Intercept)	-4.92	0.54	-5.97 – -3.86	-9.13	**<0.001**
Family size [small]	1.09	0.67	-0.23 – 2.41	1.62	0.106

Test block [2]	1.55	0.44	0.68 – 2.42	3.49	**<0.001**
Test block [3]	1.40	0.45	0.52 – 2.29	3.12	**0.002**
Age dyad [siblings]	1.30	0.25	0.81 – 1.79	5.17	**<0.001**
Novelty [Novel]	0.34	0.24	-0.14 – 0.82	1.39	0.164
Location [Vienna area]	0.58	0.36	-0.11 – 1.28	1.64	0.101
Family size [small] × Test block [2]	-1.06	0.56	-2.16 – 0.05	-1.87	0.061
Family size [small] × Test block [3]	-1.09	0.59	-2.25 – 0.06	-1.86	0.063
Family size [small] × Age dyad [siblings]	-0.39	0.40	-1.16 – 0.39	-0.98	0.328
Family size [small] × Novelty [Novel]	0.94	0.40	0.15 – 1.73	2.33	**0.020**
**Random Effects**					
σ^2	2.41				
$\tau_{00\ Dyad:Family\_Year}$	0.00				
$\tau_{00\ Family\_Year}$	0.20				
ICC	0.08				
N_{Dyad}	69				
N_{Family_Year}	11				
Observations	392				
Marginal R^2 / Conditional R^2	0.259 / 0.315				
AIC	798.215				
**Full-reduced model comparisons**					
**AIC Null model**	850.7, Chisq = 72.513, Df =10, p-value **<0.001**				
AIC Full - {Family size : Test block}	798.4, Chisq = 4.222, Df =2, p-value = 0.12				
AIC Full - {Family size : Age dyad}	797.2, Chisq = 0.97, Df =1, p-value = 0.32				
**AIC Full - {Family size : Novelty}**	801.9, Chisq = 5.675, Df =1, p-value **< 0.05**				

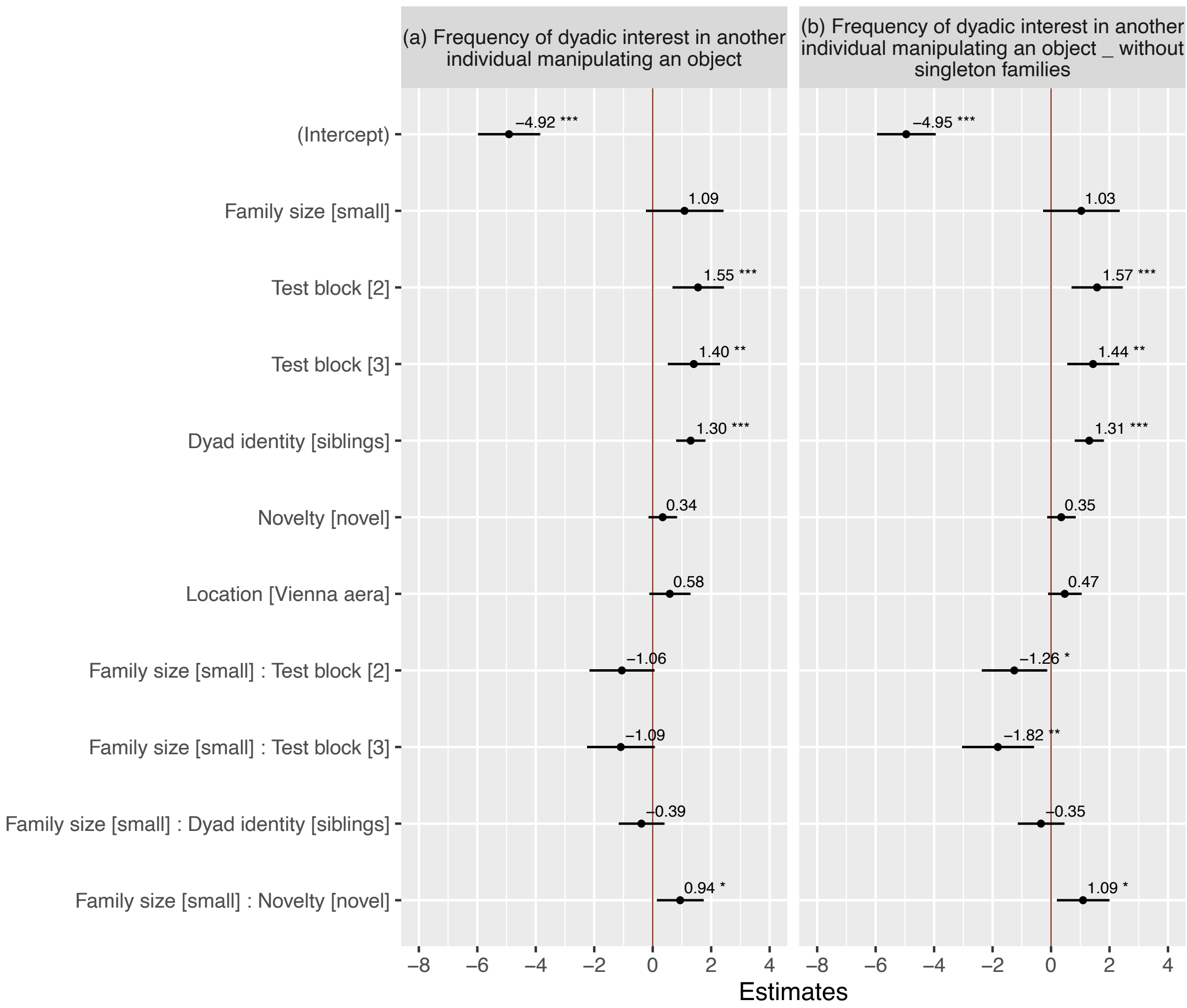

Supplementary Figure 5. Sensitivity check: comparison of estimates forest plots for the models investigating the frequency of dyadic interest by offspring in another individual manipulating an object (adult or sibling, emitted by an offspring) (a) with and (b) without singleton families (with a single chick), computed per dyad and test block. Reference levels for the categorical variables are: family size (large), test block (1), age dyad (offspring-parent), novelty (familiar), and location (Cumberland Wildpark). See electronic supplementary material, Tables S5 and S6 for the full model outputs. *** < 0.001, ** < 0.01, * ≤ 0.05.

Supplementary Table 10. Sensitivity check: GLMM results for the model investigating the frequency of dyadic interest by offspring in another individual manipulating an object (adult or sibling, emitted by an offspring), with and without singleton families (with a single chick), computed per dyad and test block. Reference levels are family size (large), test block (1), age dyad (offspring-parent), novelty (familiar) and location (Cumberland Wildpark). For each interaction term, we report the overall significance by comparison with the full model comprising the full set of predictors to a reduced model lacking the considered interaction: -{interaction term}. Comparisons were performed using a likelihood ratio test, from which we report the AIC, χ 2, degree of freedom and p-value. Significant p-values are marked in bold. *** < 0.001, ** < 0.01, * ≤ 0.05.

	**Frequency of dyadic interest in another individual manipulating an object _ without singleton families**				
Predictors	*Log-Mean*	*std. Error*	*CI*	*Statistic*	*p*
(Intercept)	-4.95	0.51	-5.95 – -3.96	-9.78	**<0.001**
Family size [small]	1.03	0.66	-0.27 – 2.34	1.55	0.120
Test block [2]	1.57	0.44	0.70 – 2.44	3.55	**<0.001**
Test block [3]	1.44	0.45	0.56 – 2.32	3.20	**0.001**
Age dyad [siblings]	1.31	0.25	0.81 – 1.80	5.22	**<0.001**
Novelty [Novel]	0.35	0.24	-0.13 – 0.83	1.43	0.151
Location [Vienna area]	0.47	0.29	-0.10 – 1.03	1.62	0.105
Family size [small] × Test block [2]	-1.26	0.57	-2.37 – -0.14	-2.21	**0.027**
Family size [small] × Test block [3]	-1.82	0.62	-3.04 – -0.60	-2.91	**0.004**
Family size [small] × Age dyad [siblings]	-0.35	0.40	-1.14 – 0.45	-0.85	0.393
Family size [small] × Novelty [Novel]	1.09	0.45	0.20 – 1.99	2.40	**0.016**

**Random Effects**	
σ^2	2.58
$\tau_{00\ Dyad:Family\_Year}$	0.00
$\tau_{00\ Family\_Year}$	0.08
ICC	0.03
N_{Dyad}	65
N_{Family_Year}	9
Observations	372
Marginal R^2 / Conditional R^2	0.253 / 0.274
AIC	712.816
**AIC Null model**	764.9, Chisq = 72.113, Df =10, p-value **<0.001**
**AIC Full - {Family size : Test block}**	718.1, Chisq = 9.289, Df =2, p-value **< 0.01**
AIC Full - {Family size : Age dyad}	711.5, Chisq = 0.725, Df =1, p-value = 0.39
**AIC Full - {Family size : Novelty}**	717.2, Chisq = 6.352, Df =1, p-value **< 0.05**

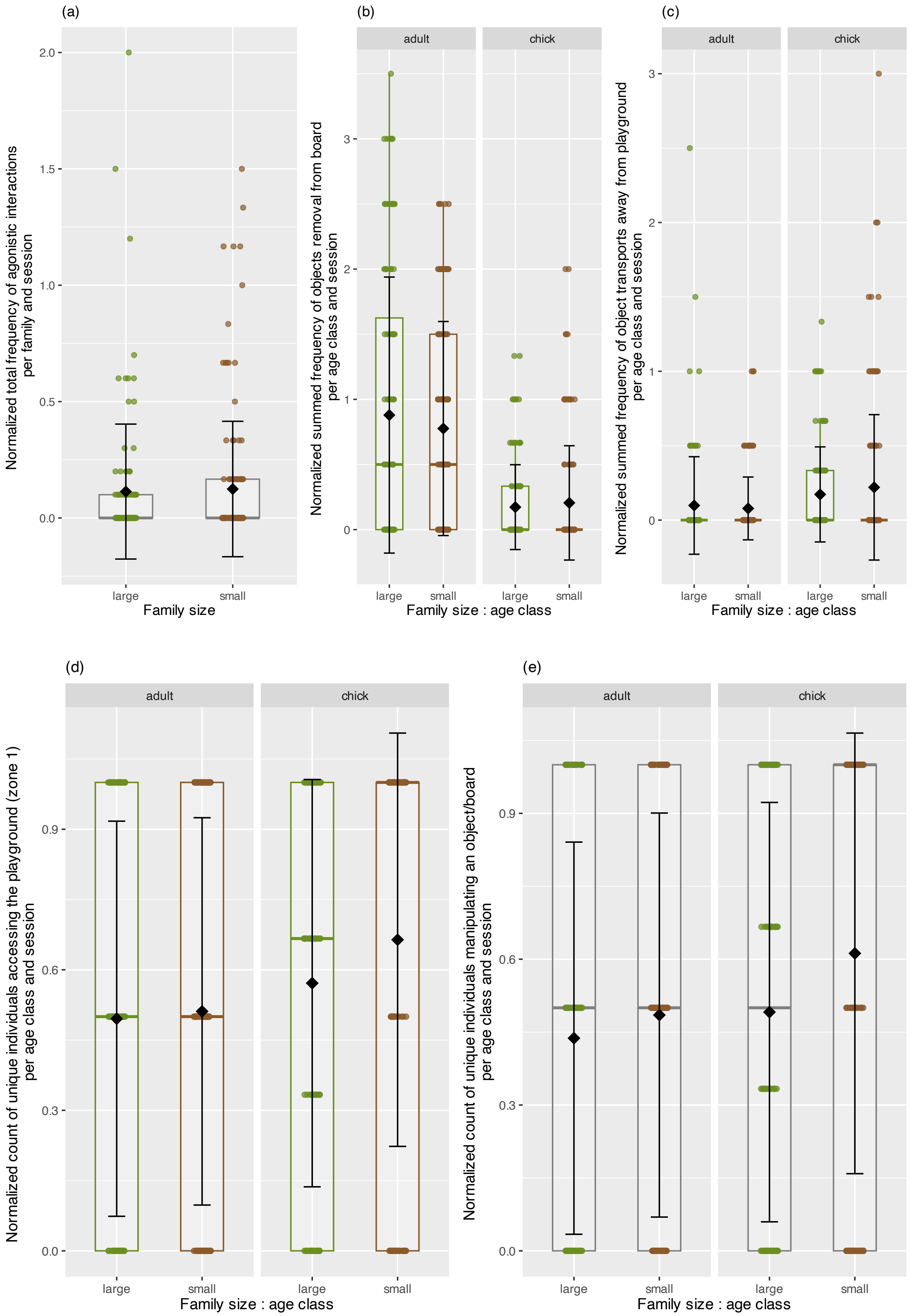

Supplementary Figure 6. Set of descriptive plots showing the effect of family size on: a) agonistic frequency; b) frequency of object removal from a board; c) frequency of object

transport away from the playground (out of zone 2); d) count of unique individuals accessing the playground; e) count of unique individuals manipulating at least once an object or a board. Across plots, data are summarised per a) family and test session, and normalised by the number of dyads in each family; c-e) age class (adult vs. chick) and test session, and normalised by the number of individuals per age class. Plots display raw data points, boxplots (showing the median and interquartile range), means (black diamonds), and standard deviations (error bars), generated using ggplot2.

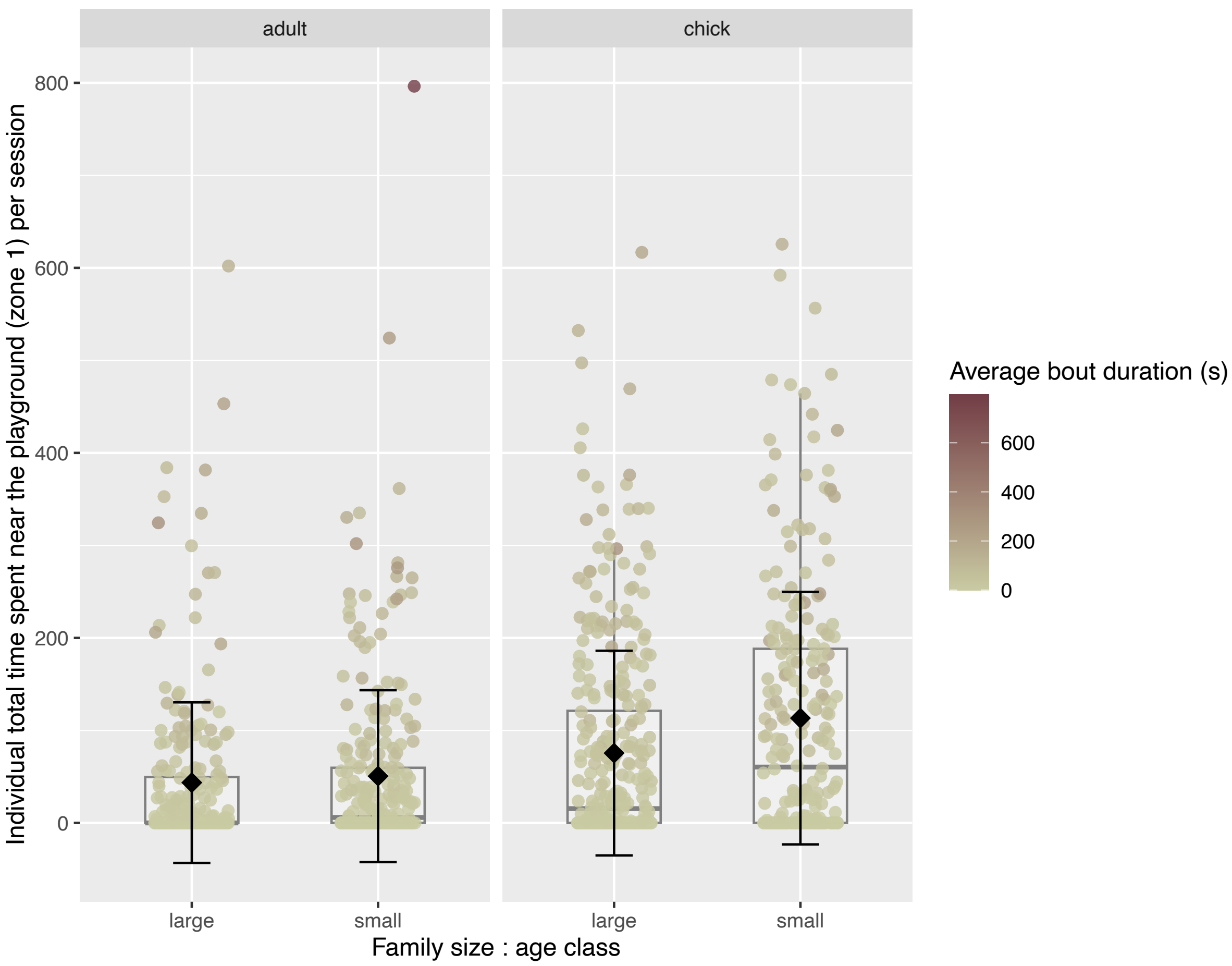

Supplementary Figure 7. Plot showing the effect of family size in interaction with individual age class (adult vs. chick) on the total time spent near the playground (zone 1) per test session. The plot displays individual raw data points, boxplots (showing the median and interquartile range), means (black diamonds), and standard deviations (error bars), generated using ggplot2.

Data points are colored along a continuous gradient representing the average bout duration per individual and test session.

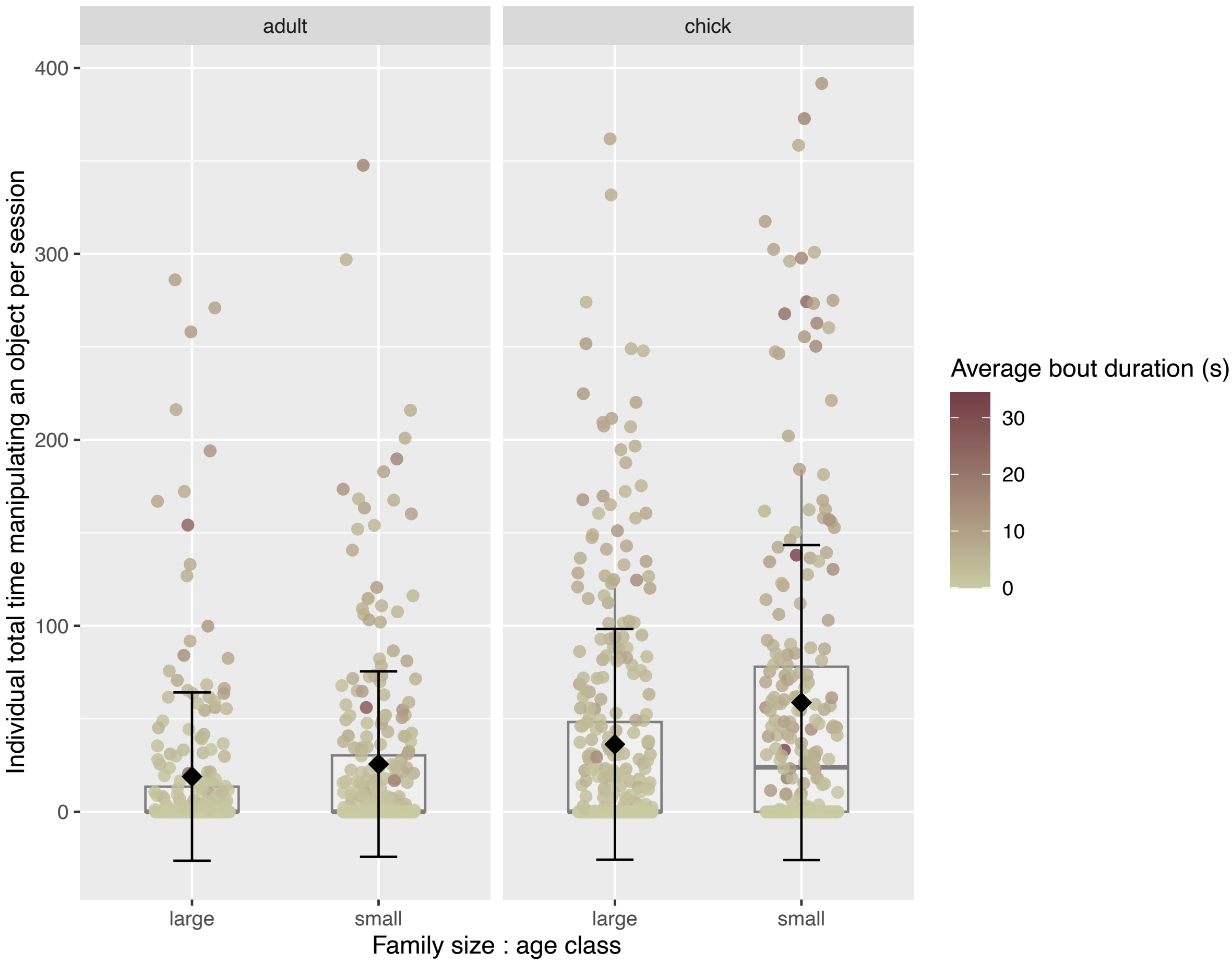

Supplementary Figure 8. Plot showing the effect of family size in interaction with individual age class (adult vs. chick) on the total time spent manipulating objects per test session. The plot displays individual raw data points, boxplots (showing the median and interquartile range), means (black diamonds), and standard deviations (error bars), generated using ggplot2. Data points are colored along a continuous gradient representing the average bout duration per individual and test session.

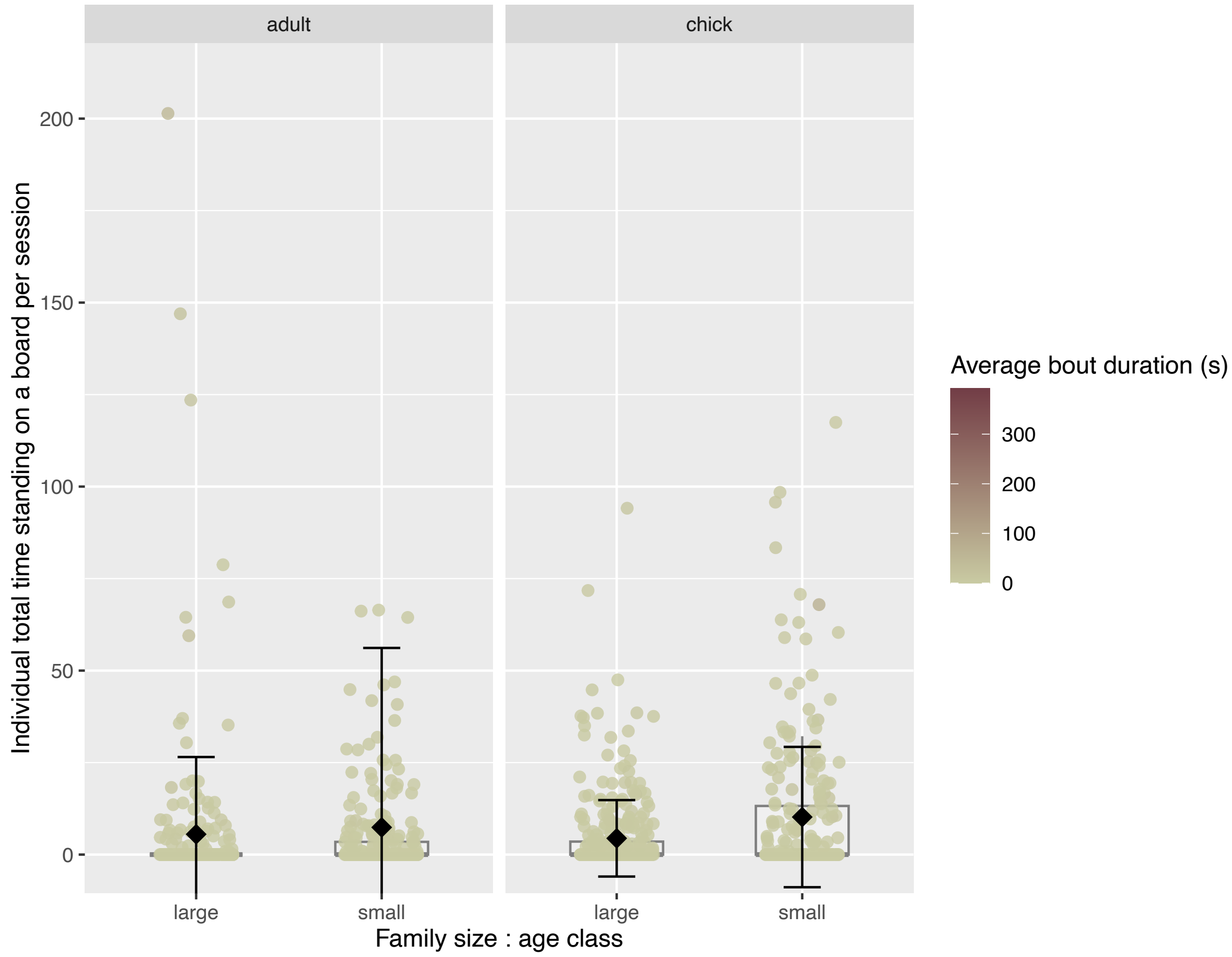

Supplementary Figure 9. Plot showing the effect of family size in interaction with individual age class (adult vs. chick) on the total time standing on a board per test session. The plot displays individual raw data points, boxplots (showing the median and interquartile range), means (black diamonds), and standard deviations (error bars), generated using ggplot2. Data points are colored along a continuous gradient representing the average bout duration per individual and test session. To improve visual clarity in panel b), axis limits were adjusted using the coord_cartesian function, which modifies the display range without excluding any data points (adult, small family, normalized total duration standing on board = 783,08).

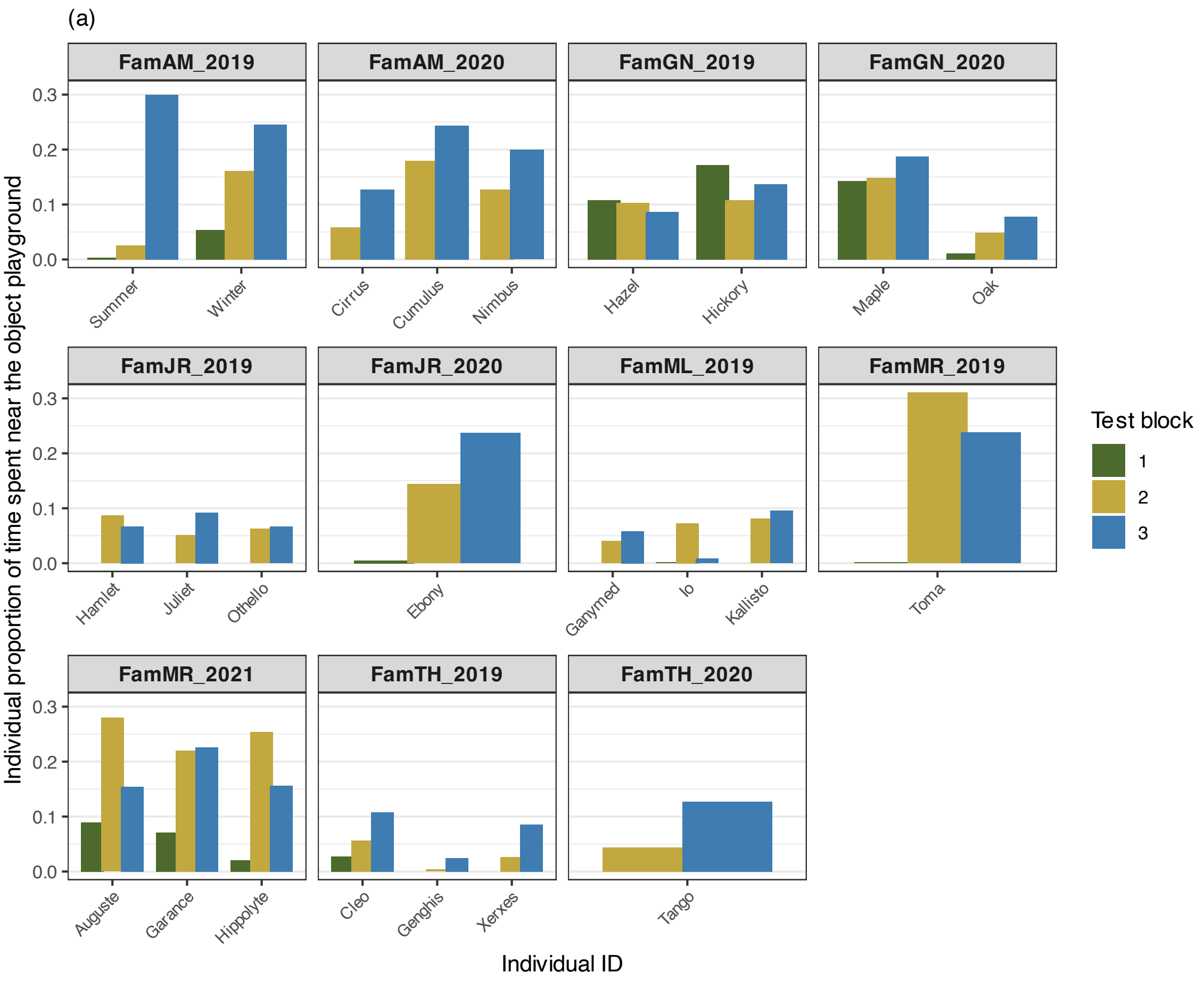
(a)
FamAM_2019
FamAM_2020
FamGN_2019
FamGN_2020
FamJR_2019
FamJR_2020
FamML_2019
FamMR_2019
FamMR_2021
FamTH_2019
FamTH_2020
0.0
0.1
0.2
0.3
Summer
Winter
Cirrus
Cumulus
Nimbus
Hazel
Hickory
Maple
Oak
Hamlet
Juliet
Othello
Ebony
Ganymed
Io
Kallisto
Toma
Auguste
Garance
Hippolyte
Cleo
Genghis
Xerxes
Tango
Individual proportion of time spent near the object playground
Individual ID
Test block
1
2
3

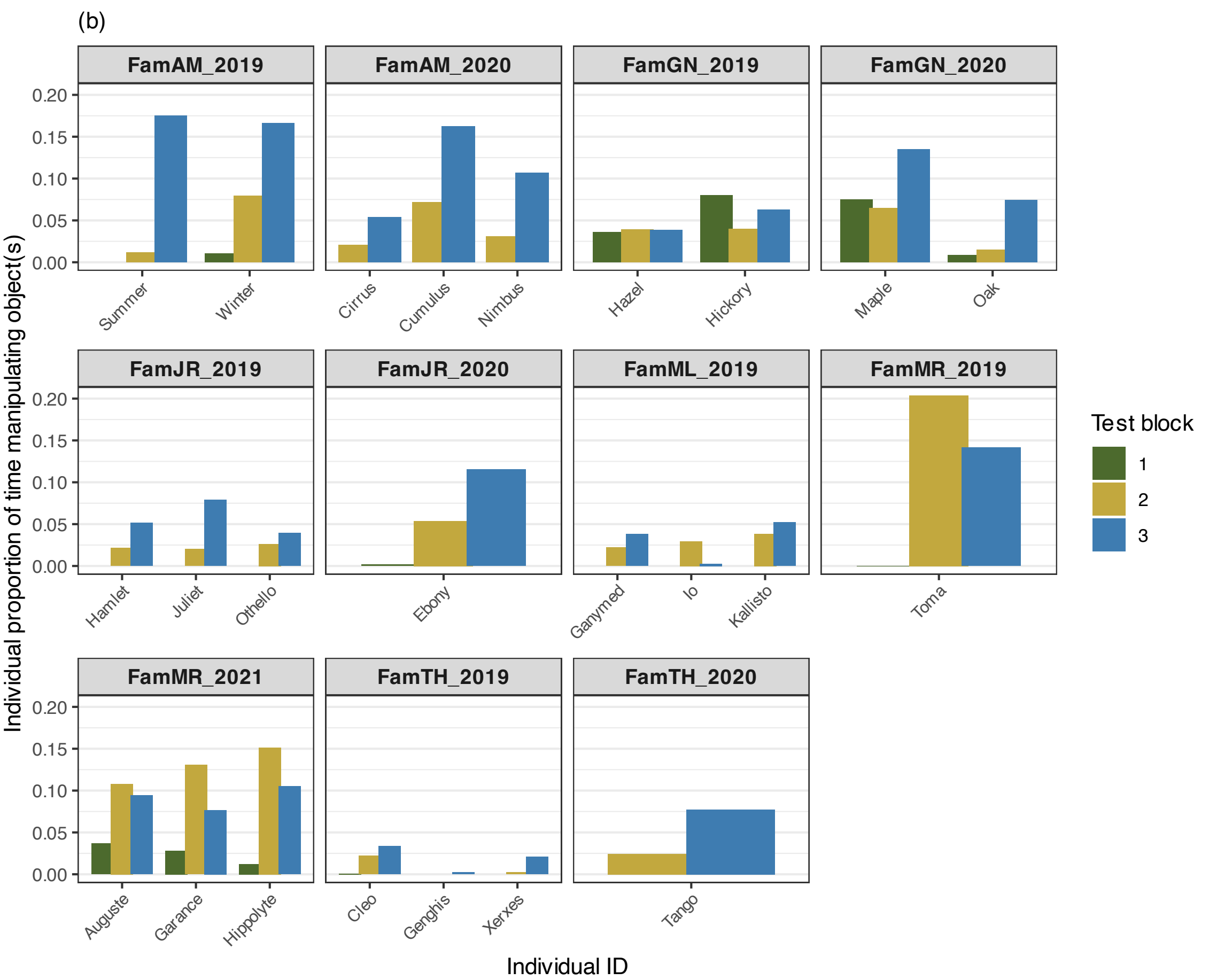
(b)
FamAM_2019
FamAM_2020
FamGN_2019
FamGN_2020
FamJR_2019
FamJR_2020
FamML_2019
FamMR_2019
FamMR_2021
FamTH_2019
FamTH_2020
0.00
0.05
0.10
0.15
0.20
Summer
Winter
Cirrus
Cumulus
Nimbus
Hazel
Hickory
Maple
Oak
Hamlet
Juliet
Othello
Ebony
Ganymed
Io
Kallisto
Toma
Auguste
Garance
Hippolyte
Cleo
Genghis
Xerxes
Tango
Individual proportion of time manipulating object(s)
Individual ID
Test block
1
2
3

Supplementary Figure 10. Series of histograms showing (a) the individual proportion of time spent near the playground or (b) manipulating objects, per individual (for offspring only) and test block. Data are aggregated per family. Plots were generated using ggplot2. The bar width fluctuations across the family grid are simply a function of the available space per facet, which changes depending on the number of individuals in each family.
