# Supplementary material for: Subtle differences in family size mediate object play in young ravens (Corvus corax)
Source: Commun Biol. 2026 Jul 11;9:1460. doi: 10.1038/s42003-026-10290-0 (PMC13737195; doi:10.1038/s42003-026-10290-0)
Supplement: Supplementary file 2 [file 42003_2026_10290_MOESM2_ESM.pdf]

nature portfolio

Corresponding author(s): Palmyre H. Boucherie

Last updated by author(s): 15/04/2026

Reporting Summary

Nature Portfolio wishes to improve the reproducibility of the work that we publish. This form provides structure for consistency and transparency in reporting. For further information on Nature Portfolio policies, see our Editorial Policies and the Editorial Policy Checklist.

Statistics

For all statistical analyses, confirm that the following items are present in the figure legend, table legend, main text, or Methods section.

n/a	Confirmed	
☐	☒	The exact sample size (*n*) for each experimental group/condition, given as a discrete number and unit of measurement
☐	☒	A statement on whether measurements were taken from distinct samples or whether the same sample was measured repeatedly
☐	☒	The statistical test(s) used AND whether they are one- or two-sided<br>*Only common tests should be described solely by name; describe more complex techniques in the Methods section.*
☐	☒	A description of all covariates tested
☐	☒	A description of any assumptions or corrections, such as tests of normality and adjustment for multiple comparisons
☐	☒	A full description of the statistical parameters including central tendency (e.g. means) or other basic estimates (e.g. regression coefficient) AND variation (e.g. standard deviation) or associated estimates of uncertainty (e.g. confidence intervals)
☐	☒	For null hypothesis testing, the test statistic (e.g. *F*, *t*, *r*) with confidence intervals, effect sizes, degrees of freedom and *P* value noted<br>*Give P values as exact values whenever suitable.*
☒	☐	For Bayesian analysis, information on the choice of priors and Markov chain Monte Carlo settings
☒	☐	For hierarchical and complex designs, identification of the appropriate level for tests and full reporting of outcomes
☒	☐	Estimates of effect sizes (e.g. Cohen's *d*, Pearson's *r*), indicating how they were calculated

Our web collection on statistics for biologists contains articles on many of the points above.

Software and code

Policy information about availability of computer code

Data collection	No software was used.
Data analysis	All our analyses were conducted in R software v. 4.4.2. with a significance threshold set at α = 0.05. The code is available as part of supplementary material.

For manuscripts utilizing custom algorithms or software that are central to the research but not yet described in published literature, software must be made available to editors and reviewers. We strongly encourage code deposition in a community repository (e.g. GitHub). See the Nature Portfolio guidelines for submitting code & software for further information.

Data

Policy information about availability of data

All manuscripts must include a data availability statement. This statement should provide the following information, where applicable:

- Accession codes, unique identifiers, or web links for publicly available datasets
- A description of any restrictions on data availability
- For clinical datasets or third party data, please ensure that the statement adheres to our policy

The datasets and R code used in this study are available as part of the electronic supplementary material.

Research involving human participants, their data, or biological material

Policy information about studies with human participants or human data. See also policy information about sex, gender (identity/presentation), and sexual orientation and race, ethnicity and racism.

Reporting on sex and gender	*Use the terms sex (biological attribute) and gender (shaped by social and cultural circumstances) carefully in order to avoid confusing both terms. Indicate if findings apply to only one sex or gender; describe whether sex and gender were considered in study design; whether sex and/or gender was determined based on self-reporting or assigned and methods used. Provide in the source data disaggregated sex and gender data, where this information has been collected, and if consent has been obtained for sharing of individual-level data; provide overall numbers in this Reporting Summary. Please state if this information has not been collected. Report sex- and gender-based analyses where performed, justify reasons for lack of sex- and gender-based analysis.*
Reporting on race, ethnicity, or other socially relevant groupings	*Please specify the socially constructed or socially relevant categorization variable(s) used in your manuscript and explain why they were used. Please note that such variables should not be used as proxies for other socially constructed/relevant variables (for example, race or ethnicity should not be used as a proxy for socioeconomic status). Provide clear definitions of the relevant terms used, how they were provided (by the participants/respondents, the researchers, or third parties), and the method(s) used to classify people into the different categories (e.g. self-report, census or administrative data, social media data, etc.) Please provide details about how you controlled for confounding variables in your analyses.*
Population characteristics	*Describe the covariate-relevant population characteristics of the human research participants (e.g. age, genotypic information, past and current diagnosis and treatment categories). If you filled out the behavioural & social sciences study design questions and have nothing to add here, write "See above."*
Recruitment	*Describe how participants were recruited. Outline any potential self-selection bias or other biases that may be present and how these are likely to impact results.*
Ethics oversight	*Identify the organization(s) that approved the study protocol.*

Note that full information on the approval of the study protocol must also be provided in the manuscript.

Field-specific reporting

Please select the one below that is the best fit for your research. If you are not sure, read the appropriate sections before making your selection.

☐ Life sciences ☒ Behavioural & social sciences ☐ Ecological, evolutionary & environmental sciences

For a reference copy of the document with all sections, see nature.com/documents/nr-reporting-summary-flat.pdf

Behavioural & social sciences study design

All studies must disclose on these points even when the disclosure is negative.

Study description	We ran quantitative analyses on behavioral data.
Research sample	We used behavioral data collected from 25 captive juvenile ravens, from 11 families between 2019 and 2021. The 11 families originated from six captive breeding pairs that reproduced over multiple years. Families included parents and their offspring (between 1 and 3).
Sampling strategy	No sample size calculation was performed.
Data collection	Experiments were conducted by groups of students from the University of Vienna (see the acknowledgement section for more details) and with the help of the animal keepers on site. All experiments were recorded using a hand-held video camera (focusing on interactions and recording verbal comments of the experimenter) and a global camera (providing an overview of the aviary) (see electronic supplementary material S1 for more details). The two different angles were combined in one single multiple-view video for scoring, using the video editing software Blackmagic Design DaVinci Resolve 16 (Blackmagic Design, 2019).We used behavioural sampling to score the 15-minute test sessions, using Loopy coding software for quantitative video analysis from Loopbio (http://loopb.io, loopbio gmbh, Vienna, Austria). We coded all individual behaviours and social interactions between family members within a 2-meter radius around the object playground. Interactions outside this zone were ignored. Additionally, we defined a smaller inner zone (zone 1), spanning 50 centimetres around the object playground, to evaluate the time individuals spent near the object set up and their proximity to one another around the playground. Note that the entire zone 1 is slightly larger than 1 meter: 50 cm on each side, plus the space occupied by the two boards (which could be moved by the ravens). For all behaviours, we coded the nature of the behaviour, the duration when relevant, and the identity of both partners for social interactions and the directionality of the interaction.
Timing	The experiment was conducted between May and June, starting as soon as the offspring had fledged (typically during the first week of May). Testing spanned three weekly blocks over nine weeks, allowing us to capture developmental (temporal) effects in our analyses. Each weekly block included test sessions conducted across four days, with one session in the morning and one in the afternoon, resulting in a maximum of eight test sessions per week and 24 test sessions per family per year. Due to the COVID-19

pandemic, two families in 2020 were tested for only two weeks, completing 16 test sessions as they missed the first test block. Additionally, one family missed two sessions during the first test block. Each test session lasted 15 minutes and started when the two boards containing the objects were provided as a 'playground' to the family group inside the aviary.

Data exclusions

One offspring in one family had unknown sex and was therefore excluded from two models out of four in the analyses (family composition: 2 chicks, one male and one of unknown sex; excluded from models investigating individual behaviour because they included sex as predictor).

Non-participation

This statement is not relevant.

Randomization

Following the number of offspring per year, families were characterized as large (F+: n=5) or small (F-: n=6; Supplementary Table 1). The number of offspring was manipulated by removing eggs from the nest, resulting in four eggs for F+ and two for F-. Importantly, this manipulation did not alter the relatedness among the chicks, as no new individuals were introduced into the families. In some nests, not all chicks hatched and/or survived the first days, resulting in the final numbers of one-three offspring per pair (Supplementary Table 1). Within pairs, the family size switched across the years.
Additionally, note that all studied proportions/frequencies were analyzed per individual/dyad and test block. In the models, family size and test block were included as a fixed effect predictor in the models. To account for non-independence of observations due to repeated individual (or dyadic) measures and to control for potential individual or dyad-level variance, we included individual or dyad identity as a random intercept. These were nested within family identity to control for shared genetic background or parental influence, in addition. For one model (assessing access to the playground), this nested structure was simplified (individual identity only) to improve model fit and avoid singularity.

Reporting for specific materials, systems and methods

We require information from authors about some types of materials, experimental systems and methods used in many studies. Here, indicate whether each material, system or method listed is relevant to your study. If you are not sure if a list item applies to your research, read the appropriate section before selecting a response.

Materials & experimental systems

n/a	Involved in the study	
☒	☐	Antibodies
☒	☐	Eukaryotic cell lines
☒	☐	Palaeontology and archaeology
☐	☒	Animals and other organisms
☒	☐	Clinical data
☒	☐	Dual use research of concern
☒	☐	Plants

Methods

n/a	Involved in the study	
☒	☐	ChIP-seq
☒	☐	Flow cytometry
☒	☐	MRI-based neuroimaging

Animals and other research organisms

Policy information about studies involving animals; ARRIVE guidelines recommended for reporting animal research, and Sex and Gender in Research

Laboratory animals

Adult and juveniles common ravens (Corvus corax), males and females. Juveniles are aged between 1,5 and 3,5 month old.

Wild animals

The study involved no wild animals.

Reporting on sex

Sex was assessed using analyses of blood sample collected on offspring when they were ringed. We worked with a total of 6 adult males, 6 adult females, 12 juvenile females and 12 juvenile males. Individual-based models included individual sex in predictors (male, female) while dyadic-based models included dyad age (parent-offspring, siblings).

Field-collected samples

The families were housed in separate aviaries located at three sites: a) Cumberland Wildpark, Grünau im Almtal; b) Haidlhof Research Station, Bad Vöslau and c) Tiergarten Schönbrunn, Zoo Vienna (Table 1). The aviaries at the first two sites are similar in size (about 80 m²); the aviary at Zoo Vienna is about double the size (~160 m²). All aviaries were enriched with wood and metal perches, trees and rocks, pool, gravel, grass, ropes, small toys and other objects (e.g., shoes, empty bottles). Birds were provided water ad libitum, and food twice a day in the morning and in the afternoon. Meals comprised meat and fresh products (e.g., fruits, vegetables, raisins, grains). For individual identification, all ravens were marked with coloured leg rings.

Note that we added the location of the aviary (Cumberland Wildpark, Vienna area) as a control fixed factor in all statistical models. Because only a single family was housed at Zoo Vienna, limiting within-site replication, we combined this location with the nearby Haidlhof research station under a common "Vienna valley" category to prevent model singularity. Both sites experience comparable climatic conditions, distinct from the alpine environment of the Cumberland Wildpark. Importantly, all facilities applied consistent standards for animal care, housing, and experimental procedures. The birds were also similarly habituated to human presence across sites, whether from researchers, students, or public visitors, helping to minimise behavioural differences related to site exposure.

Ethics oversight

The housing and testing conditions comply with the ASAB/ABS Guidelines for the Use of Animals in Research, the Austrian government guidelines, and the institutional guidelines of the University of Vienna. Permission for keeping ravens and for releasing the offspring of captive ravens into free flight was granted by the Austrian Ministry for Education, Science and Research (updated GZ

2021-0.391.938) and the BH Gmunden (Ausnahmebewilligung §29 Oö. NSchG 2001; valid from 2018-2028), respectively. The study was approved by the Animal Ethics and Experimentation Board of the Faculty of Life Science, University of Vienna (permit number 20-003). As this study was strictly non-invasive and based purely on behavioural tests, it is not classified as an animal experiment under the Austrian Animal Experiments Act (§2. Federal Law Gazette No. 501/1989). All ravens participated in the experiment voluntarily, were never deprived of food or water, and tested in their own aviaries.

Note that full information on the approval of the study protocol must also be provided in the manuscript.

Plants

Seed stocks	*Report on the source of all seed stocks or other plant material used. If applicable, state the seed stock centre and catalogue number. If plant specimens were collected from the field, describe the collection location, date and sampling procedures.*
Novel plant genotypes	*Describe the methods by which all novel plant genotypes were produced. This includes those generated by transgenic approaches, gene editing, chemical/radiation-based mutagenesis and hybridization. For transgenic lines, describe the transformation method, the number of independent lines analyzed and the generation upon which experiments were performed. For gene-edited lines, describe the editor used, the endogenous sequence targeted for editing, the targeting guide RNA sequence (if applicable) and how the editor was applied.*
Authentication	*Describe any authentication procedures for each seed stock used or novel genotype generated. Describe any experiments used to assess the effect of a mutation and, where applicable, how potential secondary effects (e.g. second site T-DNA insertions, mosiacism, off-target gene editing) were examined.*
